# Comparative genomics reveals phylogenetic distribution patterns of secondary metabolites in *Amycolatopsis* species

**DOI:** 10.1186/s12864-018-4809-4

**Published:** 2018-06-01

**Authors:** Martina Adamek, Mohammad Alanjary, Helena Sales-Ortells, Michael Goodfellow, Alan T. Bull, Anika Winkler, Daniel Wibberg, Jörn Kalinowski, Nadine Ziemert

**Affiliations:** 1 Interfaculty Institute of Microbiology and Infection Medicine Tübingen, Microbiology/Biotechnology, University of Tübingen, Tübingen, Germany; 2 German Centre for Infection Research (DZIF), Partner Site Tübingen, Tübingen, Germany; 3 School of Biology, Newcastle University, Ridley Building 2, Newcastle upon Tyne, NE1 7RU UK; 4 School of Biosciences, University of Kent, Canterbury, CT2 7NJ UK; 5 Universität Bielefeld, Center for Biotechnology (CeBiTec), Bielefeld, Germany

**Keywords:** *Amycolatopsis*, Genome mining, Comparative genomics, Biosynthetic gene cluster, Gene cluster family, Secondary metabolite diversity, Phylogeny, Natural products, Evolution

## Abstract

**Background:**

Genome mining tools have enabled us to predict biosynthetic gene clusters that might encode compounds with valuable functions for industrial and medical applications. With the continuously increasing number of genomes sequenced, we are confronted with an overwhelming number of predicted clusters. In order to guide the effective prioritization of biosynthetic gene clusters towards finding the most promising compounds, knowledge about diversity, phylogenetic relationships and distribution patterns of biosynthetic gene clusters is necessary.

**Results:**

Here, we provide a comprehensive analysis of the model actinobacterial genus *Amycolatopsis* and its potential for the production of secondary metabolites. A phylogenetic characterization, together with a pan-genome analysis showed that within this highly diverse genus, four major lineages could be distinguished which differed in their potential to produce secondary metabolites. Furthermore, we were able to distinguish gene cluster families whose distribution correlated with phylogeny, indicating that vertical gene transfer plays a major role in the evolution of secondary metabolite gene clusters. Still, the vast majority of the diverse biosynthetic gene clusters were derived from clusters unique to the genus, and also unique in comparison to a database of known compounds. Our study on the locations of biosynthetic gene clusters in the genomes of *Amycolatopsis*’ strains showed that clusters acquired by horizontal gene transfer tend to be incorporated into non-conserved regions of the genome thereby allowing us to distinguish core and hypervariable regions in *Amycolatopsis* genomes.

**Conclusions:**

Using a comparative genomics approach, it was possible to determine the potential of the genus *Amycolatopsis* to produce a huge diversity of secondary metabolites. Furthermore, the analysis demonstrates that horizontal and vertical gene transfer play an important role in the acquisition and maintenance of valuable secondary metabolites. Our results cast light on the interconnections between secondary metabolite gene clusters and provide a way to prioritize biosynthetic pathways in the search and discovery of novel compounds.

**Electronic supplementary material:**

The online version of this article (10.1186/s12864-018-4809-4) contains supplementary material, which is available to authorized users.

## Background

The value of bacterial secondary metabolites for medical applications, as pharmaceuticals, especially anti-infectives, but also for industrial use is indisputable [1, 2]. Furthermore, the demand for the discovery of novel compounds for medical applications is urgent, especially in the light of the increasing antibiotic resistance to drugs currently in use [3]. To facilitate the discovery of novel compounds, bacterial genome sequences are screened for genome regions that are likely to code for the production of secondary metabolites. This bioinformatics approach is the first important step in the genome mining pipeline that is necessary to guide the discovery of novel compounds [4, 5]. The secondary metabolite machinery of bacteria is mainly organized into several diverse clusters, called biosynthetic gene clusters (BGCs), which contain biosynthesis genes in close physical proximity. BGCs encoding for closely related biosynthetic pathways that produce highly similar chemical compounds are summarized under the term gene cluster families (GCFs). Polyketide synthase (PKS) and non-ribosomal peptide synthetase (NRPS) gene clusters are huge megasynthases that produce natural products by a multimodular assembly line in a series of chemical condensation reactions [6]. Other notable classes include ribosomally synthesized and post-translationally modified peptides (RiPPs) and terpenes [7, 8].

Recent comparative genomics approaches have shown that the potential for bacteria to produce secondary metabolites is much more promising than previously thought, as many actinobacterial genomes harbor 20–29 BGCs on average [9]. With the currently available tools, detection of putative BGCs is fast and simple [10]. It is now feasible to detect thousands of putative BGCs. To guide the discovery of the most promising novel compounds, it is important to understand the distribution patterns of BGCs. Therefore, knowledge about the diversity, environmental distribution and phylogenetic relationships of BGCs in the context of their environmental function is paramount.

In contrast to primary metabolites, bacterial secondary metabolites are not necessary for the immediate survival of the bacterium, but are important for adaption, as well as for fitness advantages in specific natural habitats. Early hypotheses suggested that bacteria mainly produce secondary metabolites with antibiotic activity for defense purposes, more recent studies show that these secondary metabolites also play a key role as signaling molecules [11, 12]. Furthermore, they have been shown to be involved in complex mutualistic relationships in their specific environment [13]. Yet, the complex functions of secondary metabolites in their natural environment remain poorly understood.

Previous approaches to characterize secondary metabolite gene clusters used different methods to sort BGCs into related GCFs [14–16]. It was shown that on one hand BGC distribution was correlated with species phylogeny while on the other hand the vast BGC diversity could not be explained by vertical evolution. Furthermore, distinct taxa, or even distinct species, show remarkable differences in their BGCs. This leaves open questions concerning the main mechanisms for secondary metabolite evolution. Because of these taxonomic differences, it is necessary to characterize many different bacterial genera in order to evaluate the diversity of BGCs and the mechanisms leading to their diversification. This knowledge should help us to predict where to seek novel secondary metabolites, and to estimate if the search for novel producers should be based on phylogeny, geography or on specific microenvironments. Classifying GCFs enables us to further prioritize BGCs with respect to their novelty and to predict their structural scaffolds [4].

In this work, we focus on the actinomycete genus *Amycolatopsis* as a model system for an in-depth study of secondary metabolite gene clusters harbored by this genus. As of 2017, 69 different *Amycolatopsis* species have been validly named [17]. 41 genome sequences representing 28 different *Amycolatopsis* species are publicly available as complete or draft genome sequences. *Amycolatopsis* strains are ubiquitously distributed and have been isolated foremost from soil, but also from aquatic habitats, rock surfaces, and from clinical sources [18–23]. Only four *Amycolatopsis* species are known to have pathogenic properties [24, 25].

*Amycolatopsis* is already valued as a producer for the commercially used vancomycin and other glycopeptide antibiotics as well as for the production of the ansamycin rifamycin [26]. Other compounds with antibacterial, antifungal or antiviral properties that have been derived from *Amycolatopsis* strains are quartromycin [27], octacosamicin [28], chelocardin [29], kigamicin [30] and the macrotermycins A-D [31].

To explore the full potential of *Amycolatopsis* strains for the synthesis of secondary metabolites, we performed a comprehensive analysis of the secondary metabolite gene clusters in *Amycolatopsis*. We were able to elucidate the phylogenetic patterns in which biosynthetic gene clusters evolve and to reveal the huge genetic potential of members of this taxon to produce novel secondary metabolites.

## Results

In order to characterize and compare members of the genus *Amycolatopsis* and to establish their potential for biosynthesis of secondary metabolites we used 43 *Amycolatopsis* genome sequences for a comparative genomics approach. In total, 41 of the 43 strains were derived from public databases and two strains, *Amycolatopsis* sp. H5 and KNN 50.9b, were newly sequenced. This Whole Genome Shotgun project has been deposited at DDBJ/ENA/GenBank under the accession NMUL00000000 (H5) and NMUK00000000 (KNN50.9b). The version described in this paper is version NMUL01000000 for *Amycolatopsis* sp. H5 and version NMUK01000000 for *Amycolatopsis sp*. KNN50.9b. Basic data for the newly sequenced strains are given in the supplementary material (Additional file 2: Table S1).

### Characterization of the genus *Amycolatopsis*

**Fig. 1:**
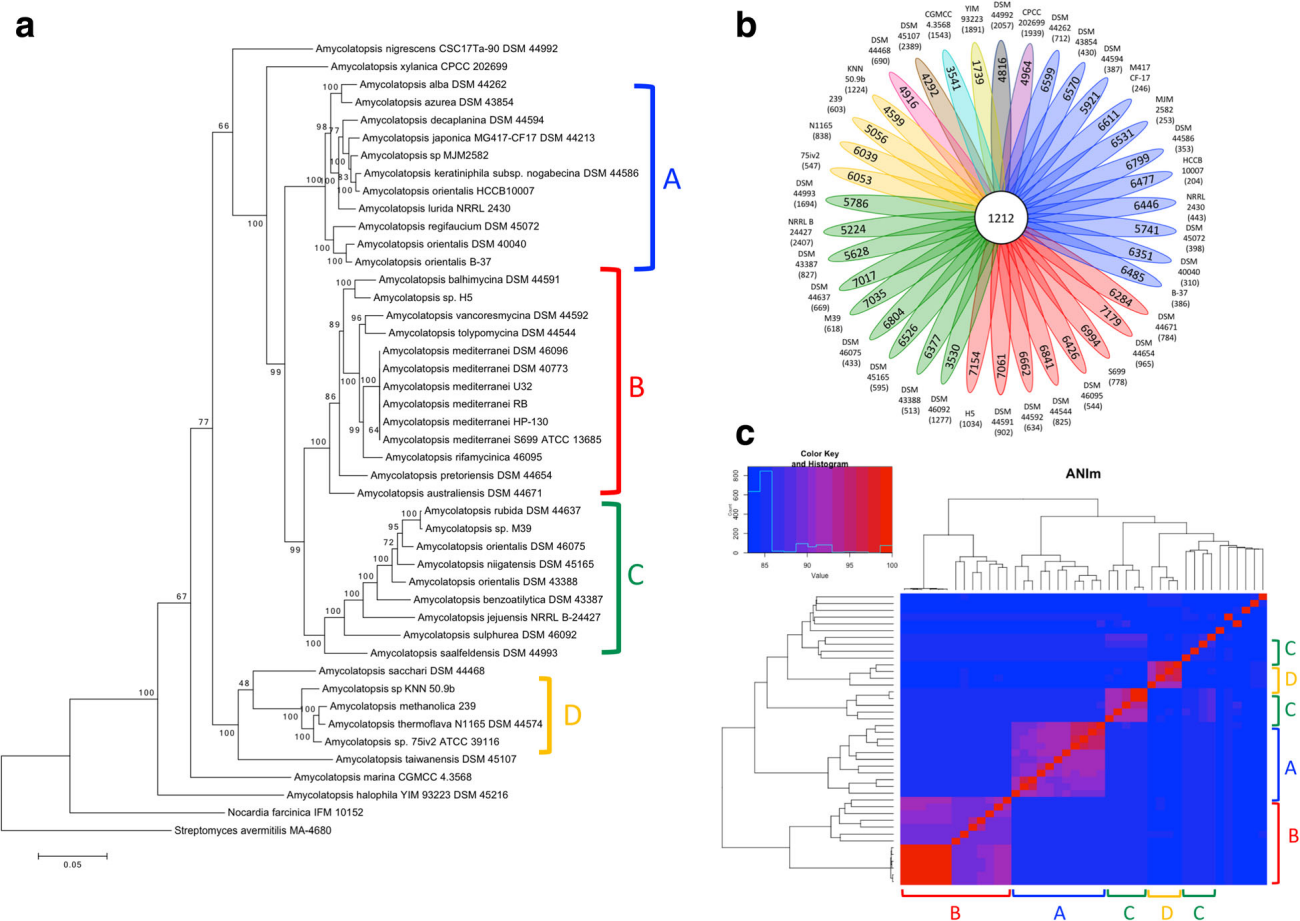
*Amycolatopsis* phylogeny, core−/pan-genome and average nucleotide identity. **a**) Maximum likelihood tree based on a MLSA (concatenated sequences of *atpD*, *clpB*, *gapA*, *gyrB*, *nuoD*, *pyrH* and *rpoB*) of 43 members of the genus *Amycolatopsis*. Bootstrap values were calculated from 500 bootstrap repetitions. **b**) Flower diagram representing the core-, accessory- and pan-genome of the *Amycolatopsis* strains. **c**) Heatmap displaying relationships between *Amycolatopsis* strains based on ANIm values

To assess relationships between the sequenced *Amycolatopsis* strains we performed a multi locus sequence analysis (MLSA). Based on the concatenation of 7 housekeeping genes (*atpD*, *clpB*, *gapA*, *gyrB*, *nuoD*, *pyrH*, *rpoB*) a maximum likelihood phylogenetic tree was generated for all of the 43 *Amycolatopsis* strains (Fig. 1a); *Nocardia farcinina* IFM10152 and *Streptomyces avermitilis* MA-4680 were used as outgroups. We were able to distinguish four major phylogenetic lineages containing the majority of the *Amycolatopsis* stains, from here on referred to as A, B, C and D. Six strains, namely *A. halophila* YIM 93223, *A. marina* CGMCC 4.3568, *A. nigrescens* DSM 44992, *A. sacchari* DSM 44468, *A. taiwanensis* DSM 45107, and *A. xylanica* CPCC 202699 formed distinct single membered clades. It was not possible to detect any significant relationships between the phylogeny of *Amycolatopsis* strains and their origin (Additional file 3: Table S2). Members from the same phylogenetic clade were isolated from various geographic regions across the world. The majority of strains were isolated from diverse soils; the marine isolate *A. marina* CGMCC 4.3568 and the salt-lake isolate *A. halophila* YIM 93223 did not clade with any of the soil strains.

Discrepancies were observed in the assignment of strains delineated as *Amycolatopsis orientalis*. Among the strains in group A is the industrial vancomycin producer *A. orientalis* HCCB10007 which clades a significant distance away from the *A. orientalis* DSM 40040 T. Furthermore, *A. orientalis* DSM 46075 and DSM 43388 fell into clade C, even further away from the *A. orientalis* type strain. When comparing the MLSA tree with a 16S rRNA tree based on sequences derived from genomic data (Additional file 1: Figure S1), similar discrepancies could be seen. *A. orientalis* HCCB10007 clades in close proximity to *A. japonica* DSM 44213, but not with the *A. orientalis* type strain DSM 40040. *A. orientalis* DSM 46075 and DSM 43388 clade with group C strains as in the MLSA tree. However, in the 16S rRNA tree it could be clearly seen that the phylogenetic resolution is too low to distinguish *Amycolatopsis* strains on a species level. One problem here is that most *Amycolatopsis* strains have multiple, in some cases different, copies of the 16S rRNA gene. While the four clades (A-D) were basically the same in the 16S rRNA tree as in the MLSA tree, in some cases the multiple 16S rRNA copies did not clade. This could be seen for example for *A. orientalis* B-37 that clades among multiple copies of *A. lurida* 16S rRNA genes, for *A. decaplanina*, which clusters with different copies of *A. keratiniphila subsp. nogabecina*, and for *A. sacchari*, which clades among *A. sulphurea* genes (Additional file 1: Figure S1).

In order to assess the genome similarity amongst the *Amycolatopsis* strains, a pan genome analysis was performed using the BPGA analysis tool [32]. To reduce any bias conferred by the 6 closely related and highly similar *A. mediterranei* genomes, only the *A. mediterranei* S699 genome was used as a reference for *A. mediterranei*. The pan-genome analysis revealed a core genome of 1212 genes with an accessory genome of 27,483 genes and 33,342 unique genes (Fig. 1b). The core-pan plot (Additional file 1: Figure S2) shows that the pan genome is likely to be extended if more genomes were added to the analysis, hence the pan genome is considered to be “open”. The core genome curve levels off, therefore the addition of more genomes to the analysis will probably not change the core genome size significantly. The COG (Clusters of Orthologous Groups) analysis (Additional file 1: Figure S3) for core, accessory and unique genes revealed that the majority of the core genes are involved in translation and ribosomal structure biogenesis. Core, accessory and unique genes are all similarly involved in transcription and amino acid transport and metabolism. A remarkable number of unique and accessory genes are involved in the biosynthesis of secondary metabolites and in transport and catabolism. The majority of genes could only be linked to some general functions or to no function at all.

As group D strains and *A. taiwanensis* and *A. halophila* were clustering apart from the majority of the strains, we suspected they might represent novel taxa, distinct from the genus *Amycolatopsis*. Consequently, the average nucleotide identity based on MUMmer (ANIm) to distinguish strains at species level, and the percentage of conserved proteins (POCP) to distinguish strains at genus level, were calculated for all vs. all strains. The results, displayed as a heatmap (Fig. 1c), show that within the phylogenetic subgroups the strains have ANIm values of 89.8–96.8% (group A), 88.7–99.9% (group B), 85.3–99.1% (group C) and 84.4–96.5% (group D). For the strains that do not clade with any of the larger phylogenetic groups the ANIm values with the other strains ranged from 83.7–84.4% (*A. nigrescens*), 83.5–85.0% (*A. xylanica*), 83.6–86% (*A. marina*) and 83.0–84.0% (*A. halophila*). Comparing these values to the average ANI observed within other bacterial genera [33] shows that all *Amycolatopsis* stains are within average boundaries specified for a bacterial genus, hence their assignment to the genus *Amycolatopsis* is supported. Results of the POCP analysis (Additional file 4: Table S3) further confirm that except for *A. halophila* all of the *Amycolatopsis* strains have at least 50% conserved proteins, and therefore belong to the same genus, while *A. halophila* might be considered a different genus.

### *Amycolatopsis* biosynthetic gene clusters - diversity and phylogenetic affiliation

To study the potential of the strains to produce secondary metabolites, all of the *Amycolatopsis* genomes were screened for candidate BGCs using the secondary metabolite identification pipeline antiSMASH. Because the estimation of precise cluster boundaries is a critical step when computationally comparing BGCs, all of the clusters detected with antiSMASH were manually curated [34]. A detailed overview on the distribution of BGCs with respect to their phylogenetic affiliation is given in Additional file 1: Figure S4.

In general, strains from the phylogenetic groups A and B have a higher number of BGCs (A: on average 37 BGCs, range 34–45 BGCs; B: on average 34 BGCs, range 28–41 BGCs) than strains from group C (on average 30 BGCs, range 22–38 BGCs). Within group D the lowest number of BGCs (on average 18 BGCs, range 14–20 BGCs) were identified. The genomes of *A. sacchari* and *A. taiwanensis*, which are distinctly related with group D, have 16 and 18 BGCs respectively. The strains from the isolated aqueous and saline environments harbor only 22 BGCs (*A. marina*) and 14 BGCs (*A. halophila*). In contrast, 43 and 41 BCGs were found in the genomes of the *A. xylanica* and *A. nigrescens* strains. When comparing the BGC representatives for the different phylogenetic clades, it can be seen that strains from groups A and B have remarkably high numbers of PKS and NRPS genes compared to the group C and D strains. The number of RiPP, terpene and other BGCs is fairly constant over the different phylogenetic subgroups, though the genome of the *A. halophila* strain lacks terpene BGCs. Overall each strain added to the analysis contributed on average 6–7 new BGCs.

The relationship between the BGCs of each *Amycolatopsis* strain was assessed by manually sorting the identified BGCs to GCFs, according to cluster architecture and Blast similarity. A concise overview of the sorting rationale is given in Additional file 1: Figure S5. Overall 442 GCFs were distinguished, the majority of which were either PKS or NRPS. It is possible to distinguish between common GCFs (present in four or more strains), rare GCFs (present in 2–3 strains) and unique GCFs (present in only one strain).

**Fig. 2:**
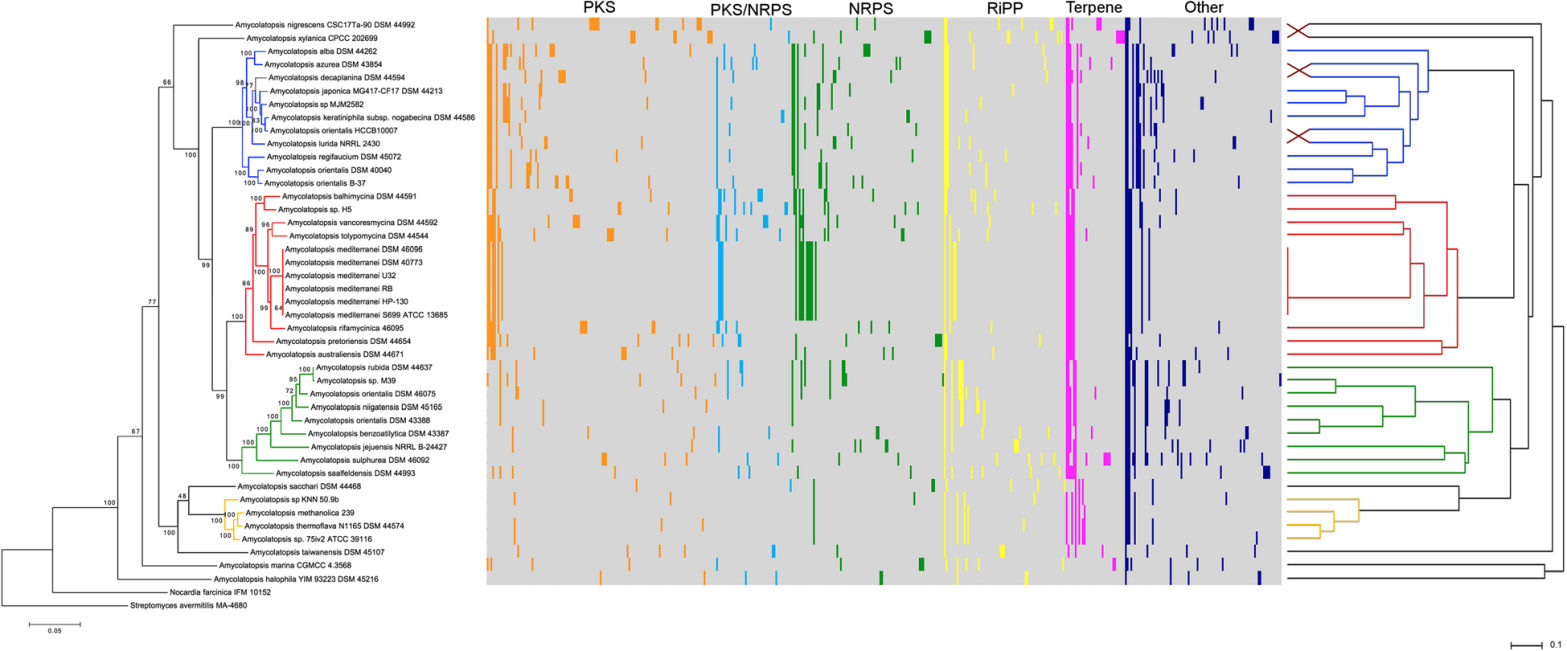
Presence/absence of GCFs in *Amycolatopsis* strains. Each column in the map stands for a gene cluster family, each row stands for a certain *Amycolatopsis* strain, respective to the phylogeny in Fig. 1a. The presence of a GCF member in a strain is highlighted by a color code according to their class: PKSs – orange, PKS/NRPS-hybrids – light blue, NRPSs – dark green, RiPPs – yellow, Terpenes – purple, all other identified BGC classes – dark blue. For each class the GCFs are sorted by abundance, from high to low abundance. The absence of the respective GCF member is shown in grey. The dendrogram (UPGMA clustering with dice similarity coefficient) is derived from a similarity matrix containing information on the presence/absence of BGCs

The distribution of GCFs amongst the members of the genus *Amycolatopsis* is visualized in Fig. 2 as a presence/absence map. It can be seen that *Amycolatopsis* strains with a high similarity in their BGC presence/absence patterns cluster together in the dendrogram. The patterns in the distribution of GCF in the main correlate with the species phylogeny. Comparing the BGCs and their phylogenetic affiliation, it can be seen that the common GCFs are usually present in all members of their phylogenetic clade and rarely cluster outside of their phylogenetic subgroups. The common PKS, NRPS and PKS/NRPS-hybrid clusters, as well as some of the RiPP families are mainly represented. Four terpene cluster families, one RiPP family and several clusters from the “others” category were present in the genomes of the majority of the *Amycolatopsis* strains. Additional file 1: Figure S6 shows the frequency of GCFs within the genus *Amycolatopsis* in detail. When comparing the distribution of GCFs, the conserved GCFs only account for a small proportion of the biosynthetic pathway diversity in *Amycolatopsis*, only 33% are rare or common GCFs. A vast number of GCFs are represented by only a single member (67% unique GCFs). The number of unique GCFs exceeds the common and occasional GCFs by a factor of two. These numbers emphasize the huge potential for strain specific diversification.

**Fig. 3:**
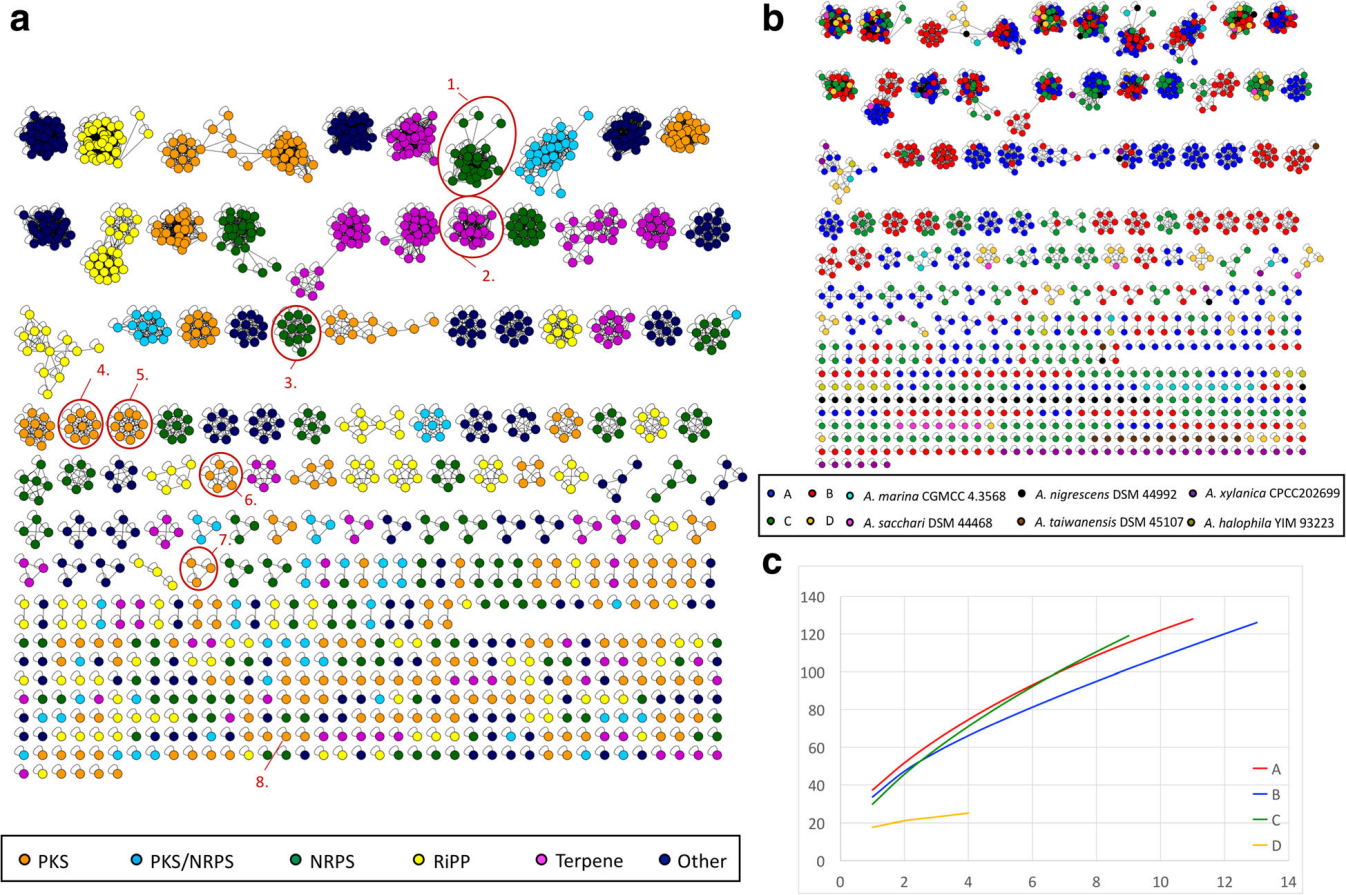
Genetic network and rarefaction curves of *Amycolatopsis* BGCs. Color codes are respective for gene cluster type (**a**) or phylogeny (**b**). A node stands for a specific BGC, while the length of the edges represents their relation, expressed through the Jaccard index value (threshold 0.65). (**c**) Rarefaction curves representing the BGC richness of the four phylogenetic subgroups. 1. albachelin-like NRPS and similar clusters (see Additional file 1: Figure S7), 2. 2-methylisoborneol, 3. glycopeptides, 4. rifamycin, 5. ECO-0501, 6. macrotermycin-like PKS clusters, 7. octacosamicin, 8. chelocardin

We also used a computational method to group BGCs into GCFs and visualized them by genetic networking. The resultant groups follow the similarity of their Pfam-domains in each cluster, as previously noted by Cimermancic et al. [16]. Using the Jaccard- and domain duplication index (DDI) as distance metrics a genetic network showing an all vs. all comparison of the *Amycolatopsis* BGCs was generated (Fig. 3a). The same color code as for the BGC-presence/absence map was used to distinguish between the BGC-classes. Most of the delineated GCFs corresponded to our previously defined GCFs. In Fig. 3a the BGCs that were previously linked to a specific secondary metabolite are highlighted. This encompasses the NRPS biosynthesis clusters encoding the albachelin and amychelin like siderophores, and the glycopeptide class of antibiotics. Furthermore, the polyketide clusters for rifamycin, ECO-0501, chelocardin and the macrotermycins are shown. The vast majority of strains harbored a 2-metyhlisoborneol encoding terpene BGC. All *Amycolatopsis* strains harbored the same ectoine BGC, which was excluded from further analyses because it should be considered as a primary metabolite. An example in which the automatically calculated GCFs differed from the manually sorted ones is shown in the Additional file 1: Figure S7.

To distinguish novel BGCs from known BGCs we used gene clusters deposited at the Minimum Information about a Biosynthetic Gene Cluster (MIBiG) database as a reference, which at the date of publication contained 1297 annotated BGCs of known compounds. A genetic network of all of the MIBiG BGCs together with all of the *Amycolatopsis* BGCs was created, using the Cimermancic index (Additional file 1: Figure S8). It was possible to distinguish 1149 clusters, 388 of which were only found in the genomes of the *Amycolatopsis* strains, 742 were MIBiG only, and 19 consisted of *Amycolatopsis* and MIBiG clusters. Of the 388 *Amycolatopsis* only clusters 275 were singletons. These results provide further evidence of the huge diversity of *Amycolatopsis* BGCs and the immense potential this genus has for the detection of novel secondary metabolites.

To estimate further relationships between the *Amycolatopsis* phylogenetic groups and the GCFs we used a different color code for the nodes in the gene cluster network, according to the strains’ phylogenetic affiliation (Fig. 3b). Of the 70 common GCFs network clusters 31 were specific for one phylogenetic group, 17 had members from two phylogenetic lineages, and 22 contained members of three or more different phylogenetic lineages. For the families with only two or three members, the numbers are too low to draw conclusions concerning the distribution of phylogenetic groups. The majority of the *A. halophila, A. nigrescens*, *A. taiwanensis and A. xylanica* BGCs remained singletons, while about half of the BGCs from *A. marina* clustered in several of the larger groups with mixed phylogeny. Some *A. sacchari* BGCs clustered with group D strains.

To assess BGC richness for a phylogenetic group a rarefaction curve, representing the abundance of BGCs per strain is shown (Fig. 3c); a steep slope of the curve indicates that it is likely that more novel BGCs will be discovered if more strains are sampled. A steep slope can be seen for all four phylogenetic groups, although that for group D is much lower. Therefore, we would expect that maximum diversity will be reached when sampling only a few more strains from group D. It can be concluded that new members of all of the phylogenetic groups have the potential to harbor yet undiscovered biosynthetic pathways. Plotting the relative number of BGCs per strain against the genome size (Additional file 1: Figure S9) revealed that phylogenetic clades A and B not only have the largest genomes but also harbor the highest number of BGCs. Members of clade C have comparably large genomes, but less BGCs while clade D strains have the smallest genomes and the lowest BGC numbers. Taken all together, the most promising phylogenetic groups for genome mining are represented by the clade A and B strains, as well as by the *A. nigrescens* and *A. xylanica* strains.

### BGC locations on the *Amycolatopsis* genomes

The relative positions of the BGCs on the genomes can provide additional information about gene transfer, rearrangements and relationships of the BGCs. As all of the *A. mediterranei* strains showed the same BGCs in the same location, this species is only represented by *A. mediterranei* strain S699 in the subsequent analyses. Since only 11 out of the 38 *Amycolatopsis* genomes were in a complete state or available as draft genome with only one scaffold we assembled the draft genomes with multiple contigs as linearized pseudo contigs. For most of the complete genomes and the pseudo contigs synteny with the respective reference strain of their phylogenetic group is given. For the *A. japonica* and *A. lurida* genomes large scale rearrangements were observed that affected the position of the BGCs.

**Fig. 4:**
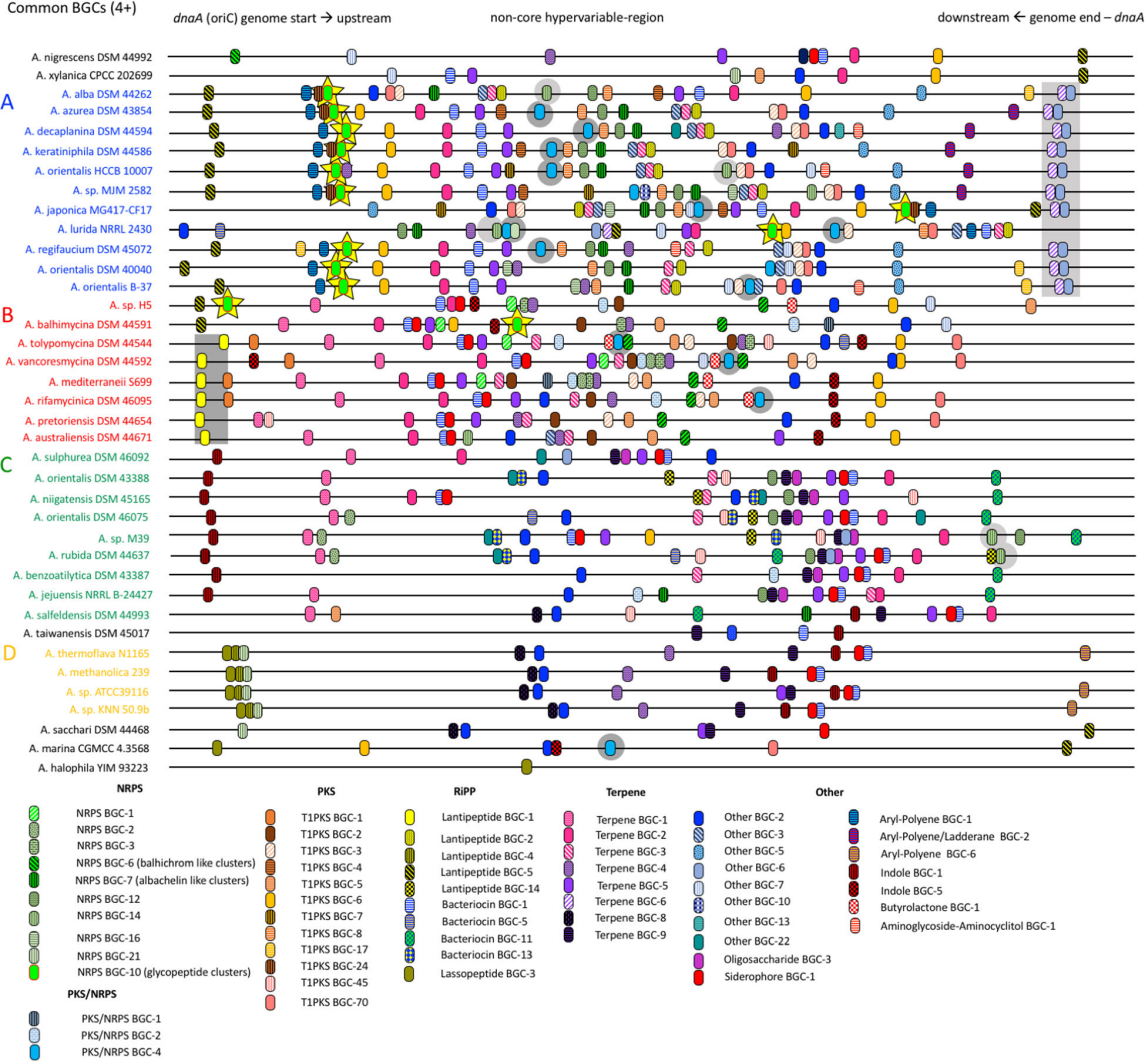
The relative location of common BGCs on linearized genomes and pseudocontigs of *Amycolatopsis*. Examples for cluster families conserved in a phylogenetic group, which also share the same location are highlighted in gray squares. Examples for cluster families with a random distribution pattern are highlighted with gray circles. The glycopeptide as example for a cluster family with unusual distribution patterns are highlighted in yellow stars

The position of each BGC was annotated on the complete genomes and pseudo contigs of all of the *Amycolatopsis* strains. Figure 4 shows the relative position of all common GCFs (with four or more members). Different patterns can be observed with respect to the distribution of BGCs throughout the *Amycolatopsis* genomes and pseudocontigs. Not only is the presence/absence of BGCs correlated with the phylogeny, but the location of most of the common BGCs is conserved within phylogenetic groups. This can be seen, for example, for “Lantipeptide BGC-1” and “Terpene BGC-6” which is always neighboring the “Other BGC-6” clusters (highlighted as grey squares in Fig. 4). For other GCFs the position on the genome is not fixed, examples are highlighted as grey circles in the Figure. This is seen best for PKS/NRPS BGC-4, which is distributed throughout phylogenetic clades A and B and is also present in the genome of *A. marina*. Another example of a BGC with a variable position is NRPS BGC-14, which is present in some members of phylogenetic clades A, B and C. Finally, an example of the huge diversity of BGCs, with respect to their locations on the genome and their phylogeny are the NRPS BGC-10 clusters, which are members of the glycopeptide family (highlighted with yellow stars in Fig. 4). All of the strains from the phylogenetic clade A and two strains from group B harbor the glycopeptide BGC in different locations on the genome. For *A. japonica* and *A. lurida* it can be speculated that the different locations on their genome is due to genome rearrangements. The presence of the glycopeptide BGCs in the group B genomes of *A. balhimycina* and in the genomes of *Amycolatopsis sp*. H5 clearly indicates that these clusters have been acquired by horizontal gene transfer (HGT).

Taken together the common BGCs tend to be located in a broad central area on the genome, opposite to the replication origin oriC, located upstream form the *dnaA* gene. These patterns can also be observed when all of the BGCs are taken into account. Additional file 1: Figure S10 shows the position of all of the BGCs on each of the linearized genomes and pseudocontigs.

**Fig. 5:**
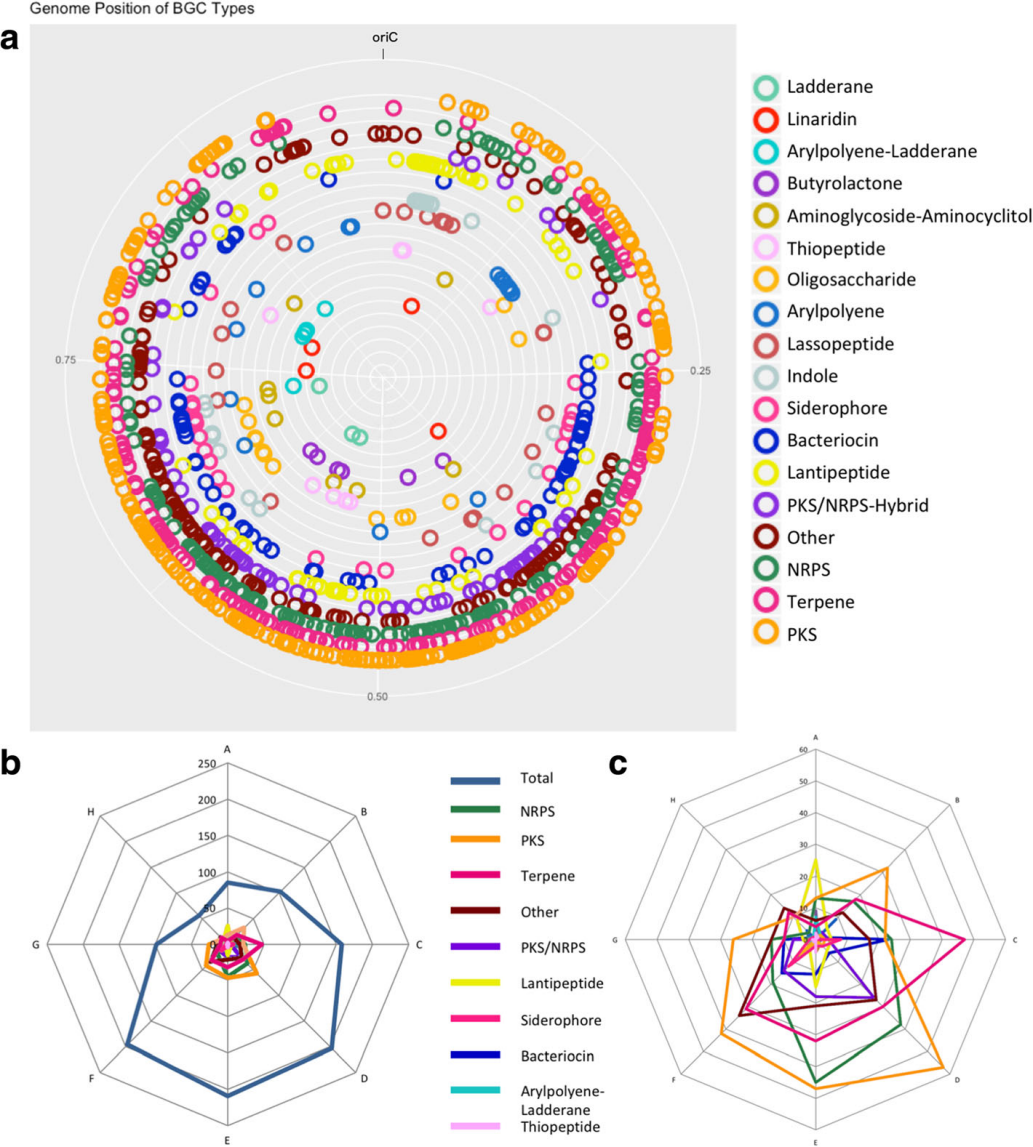
Relative location and density of all BGCs on the circular *Amycolatopsis* genomes. **a**) Relative location of *Amycolatopsis* BGCs expressed as downstream distance (0.00–1.00) to the replication origin oriC (=0.00). **b**) BGC density on certain areas of the circular *Amycolatopsis* genome (Total). **c**) BGC density on certain areas of the circular *Amycolatopsis* genome (main BGC classes)

Figure 5 shows the relative position for gene cluster types, such as terpenes, NRPS, and lantipeptides, on a circular genome model. This relative position is expressed as downstream distance (%) from oriC. For the majority of cluster types the distribution is denser around a region opposite to the replication origin, while the regions flanking the replication origin tend to have less clusters. Exceptions from these patterns are represented by the lantipeptides, lassopeptides, aryl-polyenes and indoles, where about half of the clusters are located in a region near to the replication origin.

To finally compare BGC location with overall genome conservation within the phylogenetic groups, conserved regions and hypervariable regions were identified using a PARSNP core genome alignment. Because of the large genetic differences between the *Amycolatopsis* strains, it was not possible to detect genomic islands though core-regions and hypervariable regions were observed. It can be seen that the more closely related the strains, the smaller the hypervariable regions. It can be seen from Additional file 1: Figure S11 that for the majority of BGCs the location also corresponds with the hypervariable regions of the genome.

## Discussion

Actinobacterial genome sequences have a much higher potential for the production of secondary metabolites than previously thought [35, 36]. With recent advances in bioinformatic search algorithms, it is possible to identify novel biosynthesis pathways based on predictions drawn from bioinformatics, and thereby guide the discovery of novel compounds [4]. Nevertheless, little is known about the variety and the evolutionary interconnections between secondary metabolite gene clusters and species’ phylogeny [37]. Doroghazi and Metcalf were able to portray the huge diversity of secondary metabolites in different actinomycete genera [38], but it is also apparent that the genomes of a single bacterial genus can harbor a wealth of undiscovered secondary metabolites [14, 39]. In order to study the diversity and relationships of secondary metabolites we focused on the genus *Amycolatopsis*, which is already known to produce valuable secondary metabolites [26], and to harbor a yet unknown potential for the discovery of new natural products.

To draw a comprehensive picture of the phylogenetic relations between the sequenced members of the genus *Amycolatopsis* a MLSA approach based on seven common housekeeping genes was used. At the 16S rRNA level the similarity between strains is around 97% or higher [40], hence discrimination based only on 16S rRNA data does not clearly identify relationships among members of the genus. In contrast, using MLSA, four major *Amycolatopsis* clades were detected. Furthermore, four isolates each formed a separate phylogenetic branch. By phylogenetic analysis based on 16S rRNA and an actinobacterial conserved gene, Tang et al. [41] delineated three types of *Amycolatopsis* stains: the mesophilic and moderately thermophilic *A. orientalis* clade (AOS), the mesophilic *A. taiwanensis* clade (ATS), and the thermophilic *A. methanolica* subclade (AMS). In our study we were able to further distinguish members of the AOS clade in there different phylogenetic subclades (clade A, B and C). The AMS is represented by *Amycolatopsis* group D, and the ATS clade only by *A. taiwanensis*. ANIm values underpinned these results, as ANI values within the subgroups were much higher than between them. ANI values below the 95% threshold are commonly used for species delineation [42]. On this basis, strains previously classified as *A. orientalis* HCCB10007, DSM 43388 and DSM 46075 were shown to be misclassified. No information regarding the original method of classification was available for *A. orientalis* DSM 43388 and DSM 46075. *A. orientalis* HCCB10007 was derived from the strain *A. orientalis* ATCC 43491 through physical and chemical mutageneses [43]. This strain has originally been classified *as Streptomyces orientalis*, and has since been renamed twice (*Nocardia orientalis* and *Amycolatopsis orientalis*) [20, 44]. Consequently, we agree with the previous suggestion by Jeong et al. that stains DSM 46075 and DSM 43388 belong to novel *Amycolatopsis* species [45], while further studies are needed to establish if strain HCCB10007 belongs to the species *A. keratiniphila*.

Furthermore, POCP analysis showed that *A. halophila*, which was first classified based on 16S rRNA sequencing [22], might represent a novel genus. In their study, evaluating the thresholds to define a novel genus based on the POCP values, Qin et al. suggested to consider the genome size for prokaryotic taxonomy [46]. *A. halophila* YIM 93223 also has a much smaller genome than other *Amycolatopsis* strains. Therefore, there is need to reevaluate the taxonomic status of this strain. Our results further emphasize the need to set new standards for the taxonomic classification of bacterial strains using genome sequences [47].

The majority of the *Amycolatopsis* strains were isolated from different soil types, but no correlation was found between their geographic distribution and phylogenetic relationships though the aquatic isolates, *A. halophila* and *A. marina,* did not cluster with the soil isolates. Tan et al. [48] investigated the phylogenetic diversity of different *Amycolatopsis* strains isolated from the same geographical and ecological habitat based on 16S rRNA sequencing. and showed that at the same site the strains fell into several phylogenetic groups which corresponded to the four phylogenetic subclades found in this study. Taken together these results suggest that there is no correlation between geography and phylogeny for *Amycolatopsis* soil isolates though phylogenetic diversity can be found in small, geographically close regions. The four *Amycolatopsis* sublineages are ubiquitously distributed and hence are not the consequence of adaption to a specific geographical region. In contrast, too little data are available to draw conclusions about the distribution of the aquatic isolates. Further, no correlation was found between the geographic distribution of strains and that of their BGCs, though a correlation was found between the species’ phylogeny and the distribution of BGCs. Therefore, it can be concluded that taxonomy is a more important indicator of BGC distribution than geographic origin. This phenomenon has also been observed with the marine actinobacterium *Salinispora* [14]. In general, these data support the view that geographically distant but ecologically similar habitats share overlapping gene pools. [49]. The rarefaction curves for all of the phylogenetic groups (Fig. 3c) showed that sampling more *Amycolatopsis* genomes, will lead to the discovery of novel BGCs even if the sampling was restricted to the same geographic regions and soil types.

Core−/pan-genome analysis revealed that members of the genus *Amycolatopsi*s shared a core genome of 1212 genes and a pan genome of 27,483 accessory and 33,342 unique genes. So far only few core−/pan-genome studies have been carried out for actinobacteria with comparably large genomes (5–10 Mb). A study on 17 *Streptomyces* species revealed a core genome of 2018 genes, with 11,743 in the accessory genome, and 20,831 in the unique genome [50] while another one on 31 *Streptomyces* species revealed 2048 core genes, 9806 accessory and 17,840 unique genes [51]. Similarly, a comparative genomic analysis of 17 species of the genus *Nocardiopsis* revealed a core genome of 1993 genes and a pan genome of over 22,000 genes [52]. To identify and compare ortholog clusters, these studies used the pan genome analysis pipeline PGAP [53]. A second analysis using PGAP with 37 *Amycoaltopsis* genomes showed very similar results, albeit different exact numbers (Additional file 1: Figure S12). The core/pan-genome difference between both methods can be explained by leaving out *A. nigrescens* from the analysis and by the fact that the original NCBI annotations had to be used to prepare the input data for PGAP. Both analyses reveal a very small core genome compared to other studies. It is likely that this discrepancy results from the higher number of genomes compared in our study, which usually results in a lower core genome and shows the diversity of the genus.

The *Amycolatopsis* pan-genome is quite large and is still considered as “open”. This shows that members of the genus have an extensive adaptive capacity. The COG analysis (Additional file 1: Figure S3) showed that a major part of the accessory and unique genes of the *Amycolatopsis* strains are involved in secondary metabolite biosynthesis and transport. Previous studies suggested that the diversity of secondary metabolites in bacteria is highly dependent on the bacterial genus [16, 38]. It is clear from this study that the capacity of members of the genus *Amycolatopsis* to produce diverse secondary metabolites is comparable to that of the genera *Mycobacterium* and *Streptomyces* [38].

When taking a closer look at the potential of *Amycolatopsis* strains to synthesize secondary metabolites different trends are apparent in the diversity and distribution of BGCs: I) Some BGCs were found in members of all four of the subgroups. These BGCs mainly encoded ectoines, non-NRPS derived siderophores, terpenes and RiPPs; no PKS or NPRS clusters fell into this grouping. These BGCs probably play a universal role in the metabolism of *Amycolatopsis*, and therefore might be seen as core-secondary metabolite clusters. II) In contrast, a correlation with the subgroup phylogeny was shown for most of the common BGCs. These clusters have most likely been acquired through HGT in an ancestor strain, and have been retained throughout speciation. III) The extensive range of unique BGCs observed accounted for 67% of the diverse *Amycolatopsis* GCFs and seemed to be derived from recent HGT events. These clusters might be retained, if they enhance the ability of strains to colonize ecological niches, or might be lost, and/or replaced if no such advantage is realized [37].

Two previous studies on the diversity of secondary metabolites within actinobacterial taxa gave contradictory results on the relationship between phylogeny and diversity of BGCs. Doroghazi et al., found that in 860 actinobacterial genomes BGC diversity for PKS and NRPS genes correlated with phylogeny at the species level thereby revealing the importance of secondary metabolites for speciation [15]. In contrast, Cimermancic et al. reported that the highest BGC diversity was at the tips of phylogenetic trees, indicating that their diversification is phylogeny independent [16]. BGC diversity in the present study reflects both of these trends suggesting that vertical gene transfer might be the most important driver for the maintenance of common BGCs while recent HGT events independent of phylogeny, as seen as through the singletons and, phylogenetically independent cluster families might lead to further diversification. The tendency of phylogenetically related BGCs to be located at the same position in the genomes of *Amycolatopsis* supports the hypothesis that these BGCs may have arisen from the same ancestral strain. At the same time the observation that BGCs which belong to the same cluster family are present in distinctly related strains is in line with their distribution by HGT.

Previous studies on the diversity and evolution of *Salinispora* BGCs showed that a number of BGCs was fixed over globally distributed populations [54], though the highest diversity of *Salinispora* BGCs by far were derived from unique BGCs, on average 1–2 were found even within highly conserved species [14].

Similar observations to those outlined above can be made for *Amycolatopsis* where BGC diversity is derived mostly from singleton BGCs. As *Amycolatopsis* strains are not as closely related to one another as *Salinispora* strains, an average of 6–7 novel BGCs tend to be present in new species though BGC fixation beyond the species level was observed within the phylogenetic subgroups.

The majority of BGCs in *Amycolatopsis* genomes tend to be located in a region opposite the core region surrounding the origin of replication. This suggests that the acquisition of BGCs via HGT occurs preferentially in non-core regions of the genome. The distinction between core- and non-core-regions has previously been proposed for the genomes of *A. mediterranei* U32 [55], *A. orientalis* HCCB10007 [43] and *A. methanolica* 239 [41], where regions with a lower density of coding genes were observed and considered to be non-core-regions. In general, these regions correspond with the regions of high BGC diversity observed in the present study although the proposed variable regions are larger than the non-core-regions proposed for strains U32, HCCB10007 and 239. A similar phenomenon has been observed for *Streptomyces* where a core region in the linear chromosome around the replication origin is conserved, while the arms of the chromosome display a high variability and contain the majority of species specific sequences [56]. In this same study, it was also reported that the more phylogenetically distant the strains, the greater the size of the variable region. In the present study, it was found that within the closely related subgroups (groups A, B and D) the size of the hypervariable region opposite the *dnaA* gene is smaller than in the distantly related subgroup (group C). All in all, our study is in agreement with the hypothesis that BGCs are located mainly in the non-core region, probably because insertions in essential gene clusters would in most cases prove to be lethal for the organism [56]. However, the fact that some BGCs, such as these coding for lantipeptides, are mainly located in the core region shows that BGC-location is not exclusively found in the hypervariable regions indicating that insertions in core regions are not necessary lethal.

In the present study it was not possible, as is the case of the more highly conserved genus *Salinispora* [57], to detect precise genomic islands, given the extreme genetic variation and small core genome though hypervariable regions were evident within the genetic subgroups. These hypervariable regions corresponded with the majority of BGCs, but showed no consistent structural similarities, as corresponding flanking regions, or conserved mobile elements. To establish whether a “pathway swapping” mechanism, as evident for *Salinispora* [14], is also true for *Amycolatopsis*, a larger number of more closely related strains needs to be analyzed.

## Conclusions

A comparative analysis of the genus *Amycolatopsis* and its’ biosynthetic potential revealed a highly variable gene content. All of the *Amycolatopsis* strains showed a small core-genome, but had a huge pan-genome indicating a great potential for the production of secondary metabolites. We were able to distinguish four phylogenetic sublineages within the genus *Amycolatopsis*, and four strains that formed distinct lineages in the phylogenetic tree. When comparing the phylogenetic resolution with the potential of *Amycolatopsis* strains to produce secondary metabolites an extensive diversity of BGCs was seen, most of which comes from clusters unique to the genus. Horizontal and vertical gene transfer seem equally important to drive and maintain the diversity of secondary metabolites. Among the vertically inherited clusters, a few extend across several phylogenetic lineages but most are specific for individual lineages. The observation that really novel clusters acquired through HGT were detected shows that related biosynthetic pathways can be transferred to unrelated strains through this mechanism. Further, it is evident that novel BGCs are mainly, but not exclusively incorporated into non-core hypervariable regions opposite the replication origin on the circular *Amycolatopsis* genomes.

## Methods

### *Amycolatopsis* genomes

All of the *Amycolatopsis* genome sequences available in December 2016 at the National Center for Biotechnology Information (NCBI) database [58] and the DOE Joint Genome Institute -Integrated Microbial Genomes & Microbiomes (JGI-IMG) database [59], were used. Draft genomes that consisted of more than 300 contigs and sequences from single cell genomic approaches were omitted due to quality issues.

For the sequencing of the *Amycolatopsis* sp. H5 and KNN 50.9b genomes, sequencing libraries were prepared by applying Illumina TruSeq DNA PCR-Free Library Preparation Kits with a target insert size of 550 bp. Subsequent paired-end sequencing was performed on an Illumina HiSeq 1500 System (Illumina, San Diego, CA, USA) using HiSeq Reagent v3 Kits (Illumina, San Diego, CA, USA). Read length was 2× 250 bp. Base calling was performed with an in-house software platform [60]. To assemble the resultant reads, the gsAssembler software (Newbler) v2.8 was used. The genome sequence was submitted to the NCBI Prokaryotic Gene Annotation Pipeline for annotation.

### Comparative analysis of *Amycolatopsis* strains

To elucidate the phylogenetic relationships between the *Amycolatopsis* strains a multilocus sequence typing approach based on the concatenation of seven housekeeping genes *atpD*, *clpB*, *gapA*, *gyrB*, *nuoD*, *pyrH* and *rpoB* was used. The single gene sequences were aligned using ClustalW, embedded in MEGA6.0 software [61], trimmed with respect to the reading frame and subsequently concatenated with the FaBox Fasta Alignment Joiner [62]. A maximum likelihood tree was generated using the Tamura-Nei Model with NNI (Nearest Neighbor Interchange) and 500 bootstrap replications was calculated with MEGA6.0 software.

Core−/pan-genome analysis was performed using the Bacterial Pan Genome Analysis (BPGA) tool [32]. To avoid bias derived from different annotations all of the genome sequences were newly annotated using PROKKA 1.2 with default settings [63]. As all six of the *A. mediterranei* genomes were highly similar *A. mediterranei* S699 was taken to represent the species to avoid bias. Orthologous genes were identified with the USEARCH algorithm [64] using a threshold of 0.5. Variations of the similarity threshold to 0.3, 0.4, 0.6 an 0.7 did not significantly alter the results, therefore the default threshold of 0.5 was chosen. Core−/pan-genome plots were calculated over 500 iterations. For comparative purposes an additional core−/pan-genome analysis was performed using the pan genome analysis pipeline PGAP [53]. Runs were performed using default settings under the MP and GF mode of PGAP.

To resolve the relationship of *Amycolatopsis* strains on the genus and species level the percentage of conserved proteins (POCP) was calculated as previously described [46], and the Average Nucleotide Identity based on the MUMmer algorithm (ANIm) was calculated with JSpecies using the default settings [65]. Graphical visualization of ANIm values was implemented with R version 3.3.3 [66].

### BGC and GCF identification

The biosynthetic gene clusters of all of the *Amycolatopsis* strains were identified using antiSMASH 3.0 with default settings [10]. Identified clusters were compared using MultiGeneBlast [67]. Cluster boundaries were determined as previously described [34] and clusters were manually trimmed using Artemis [68].

Assigning gene clusters to GCFs was based on manual inspection of the antiSMASH output files, a comparison with multigeneblast and sequence comparison of KS and C domains was achieved using BLAST [69] and NaPDoS [70]. The following criteria had to be met for BGC clusters to be assigned to the same gene cluster family: I) The gene clusters had to have a similar architecture, II) The majority of genes included in the cluster needed to have the same function, but not necessarily in the same order. III) The majority of genes in the genome needed to have a BLAST similarity of at least 50% identity over an 80% coverage rate. IV) For modular PKS, NRPS and their hybrid clusters a BLAST similarity of the respective KS and C domains was considered. Hence, KS and C domains with the same modular position in the different clusters were compared. Clusters where the majority of KS and/or C domains shared a BLAST identity over 80% were considered to belong to the same GCF. Results were collected in a presence/absence matrix, with 1 representing the presence and 0 the absence of a GCF member in each of the *Amycolatopsis* strains. Hierarchical cluster analysis using the DICE coefficient with UPGMA (Unweighted Pair Group Method with Arithmetic mean) was performed with PAST [71]. Comparison of the *Amycolatopsis* phylogenetic tree with the BGC-dendrogram was performed with Dendroscope v3.5.7, using the Tanglegram algorithm [72].

For genetic networking, the Pfam-domains of each BGC were identified using HMMER 3.1b2 [73] with the respective Hidden Markov Models (HMM) obtained from the Pfam database [74]. A similarity index based on the absence or presence of Pfam domains was used to delineate BGC similarity, as previously described by Lin et al. [75] with the modifications of Cimermancic et al. [16]. A similarity threshold of 0.65 was chosen, because it best reflected the manually determined GCFs. The threshold was evaluated manually, as the threshold values of 0.5 [16] and 0.8 [76] described in previous publications were not found to be suitable to distinguish between the *Amycolatopsis* BGCs. The resulting similarity matrix was visualized with Cytoscape 3.4.0 [77].

Rarefaction curves displaying the relative BGC richness for each phylogenetic group were calculated from the BGC presence/absence matrix using EstimateS [78].

### BGC location

To schematically display the relative positions of the common BGC clusters on the *Amycolatopsis* genomes, the approach previously described by Ziemert et al. [14] was used. First, the draft genomes were assembled as pseudocontigs on the phylogenetically closest complete genome as a reference using CONTIGuator v2.7 [79]. The circular genomes were linearized, using the *dnaA* gene as the start for each linearized pseudocontig. If necessary, the reverse complement sequence was used for genome alignment. Second, the position of the respective BGCs on the complete genomes and on the pseudocontigs was annotated using geneious R9.1.6 [80]. Finally, the complete genomes and pseudocontigs were normalized in length to visually distinguish between the relative position of the BGCs on the genomes and pseudocontigs. The contigs were aligned to the closest related complete genome within the same phylogenetic group. The circular genomes were linearized and normalized in length. An overview of the of complete genome and pseudo contig synteny is shown in Additional file 1: Figure S13.

To distinguish conserved regions from hypervariable regions on the *Amycolatopsis* genomes and pseudocontigs, and to identify genome rearrangements, the Harvest toolkit containing the Parsnp v1.2 tool for core genome alignment and Gingr 1.2 for visualization was used [81]. Due to the small core genome of *Amycolatopsis*, a core genome alignment for all of the strains was not feasible hence, core genome alignment for the phylogenetic subgroups that shared 85% ANIm was performed. This excluded the genome sequences of *A. halophila*, *A. marina, A. nigrescens*, *A. sacchari*, *A. taiwanensis and A. xylanica* from this analysis.

BGC density plots were created with R version 3.3.3 [66]. Thereby, the genome was divided into 8 regions, and density plots were built showing the abundance of BGCs in each region, for each cluster type and for all cluster types in total.

## Additional files

Additional file 1: Supplementary Figures. **Figure S1-S13** (PDF 6871 kb)

Additional file 2: **Table S1.** Sequencing statistics for *Amycolatopsis sp*. H5 and *Amycolatopsis sp*. KNN50.9b. (DOCX 42 kb)

Additional file 3: **Table S2.** Basic features of *Amycolatopsis* genomes. (DOCX 110 kb)

Additional file 4: **Table S3.** POCP analysis. (XLSX 20 kb)

## Abbreviations

AMS: Thermophilic *Amycolatopsis methanolica* subclade
ANI: Average Nucleotide Identity
AOS: Mesophilic/moderately thermophilic *Amycolatopsis orientalis* subclade
ATS: Thermophilic *Amycolatopsis taiwanensis* subclade
BGC: Biosynthetic gene cluster
GCF: Gene cluster family
HGT: Horizontal gene transfer
MIBiG: Minimum Information about a Biosynthetic Gene cluster
NRPS: Non-ribosomal peptide synthetase
PKS: Polyketide synthase
POCP: Percentage of Conserved Proteins
RiPP: Ribosomally synthesized and post-translationally modified peptides
