# Supplementary material for: Comparative genomics reveals phylogenetic distribution patterns of secondary metabolites in Amycolatopsis species
Source: BMC Genomics. 2018 Jun 1;19:426. doi: 10.1186/s12864-018-4809-4 (PMC5984834; doi:10.1186/s12864-018-4809-4)
Supplement: Supplementary file 1 — Supplementary Figures. Figure S1-S13 (PDF 6871 kb) [file 12864_2018_4809_MOESM1_ESM.pdf]

Supplementary Figures

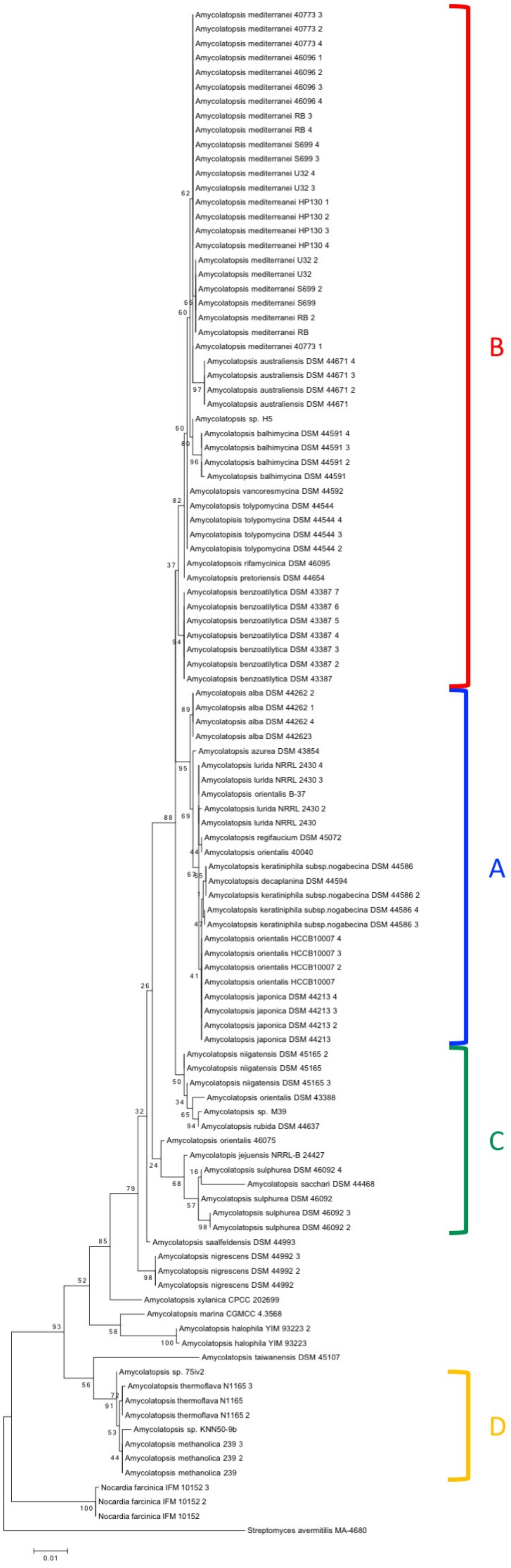

Figure S1: Maximum likelihood tree (500 bootstrap repetitions) based on a 1177 bp alignment of all complete copies of the 16S rRNA gene derived form 43 *Amycolatopsis* complete and draft genome sequences. *Nocardia farcinica* IFM 10152 and *Streptomyces avermitilis* MA-4680 were used as outgroups.

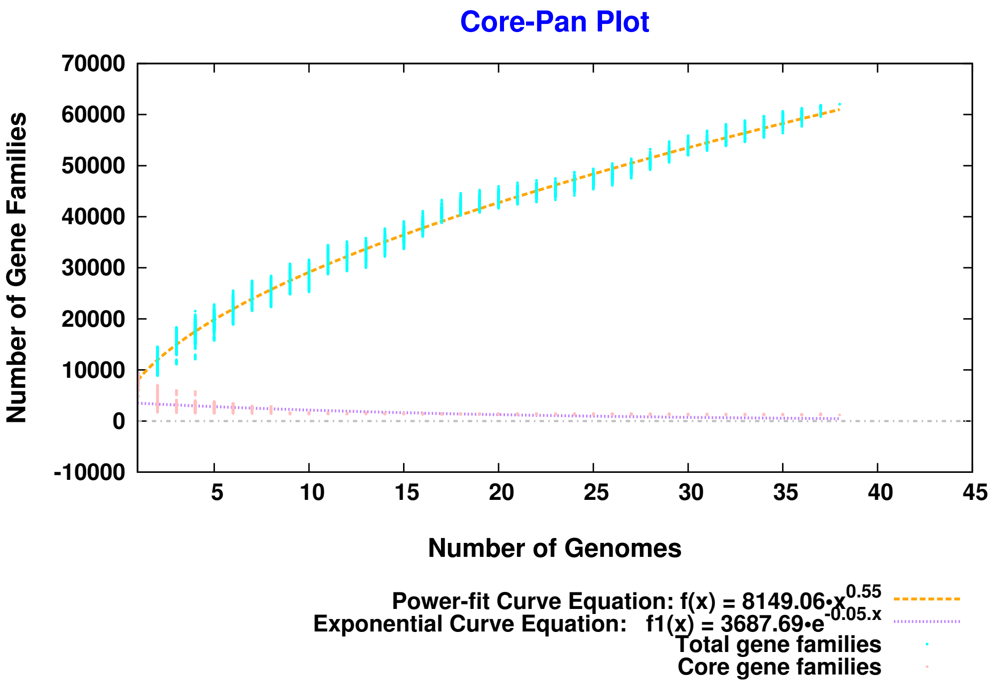

Figure S2: Core-/Pan-Genome plot for the genus *Amycolatopsis*. The number of new gene families is plotted against the number of genomes added to the analysis (orange) for the pan-genome. The number of gene families present in all strains plotted against the number of genomes (lilac). The curves are calculated based on 500 iterations with randomly changed genome order. Ranges for each added genome are displayed in cyan (pan-genome) and light pink (core genome).

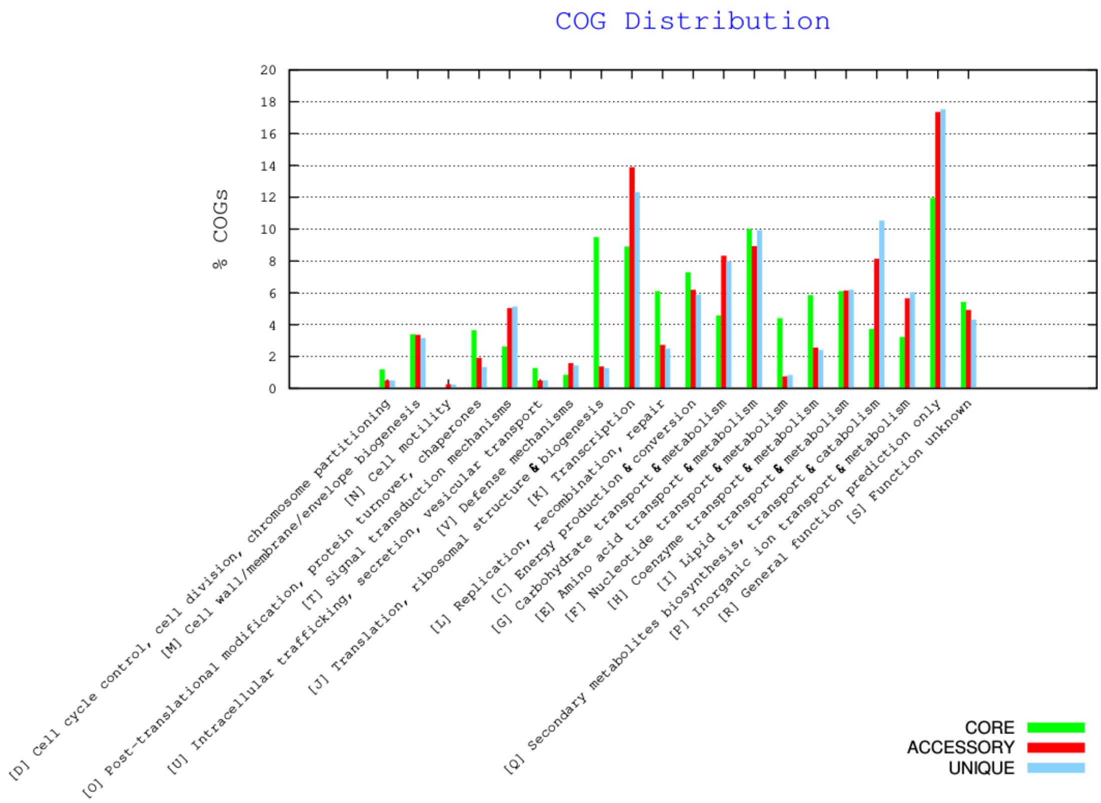

Figure S3: COG (Cluster of Orthologous Groups) distribution of core-, accessory- and unique genes in *Amycolatopsis*.

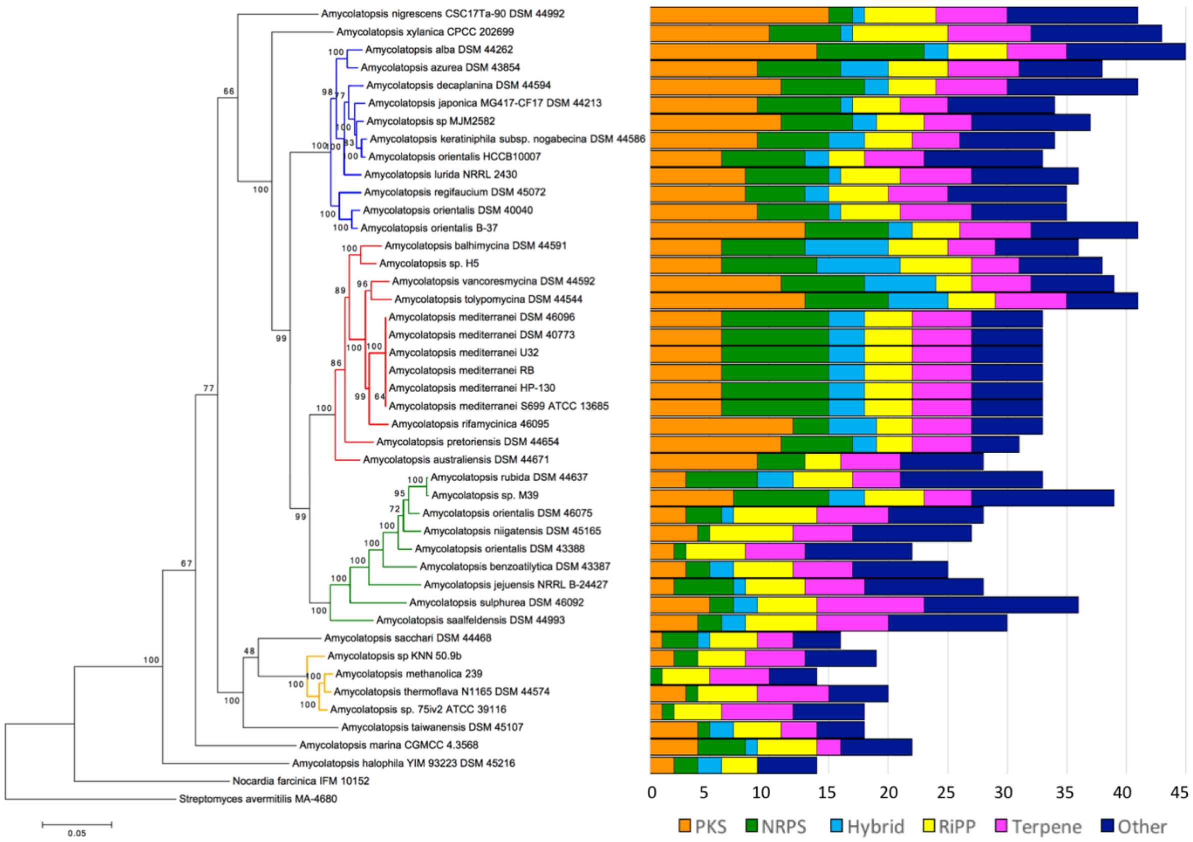

Figure S4: *Amycolatopsis* maximum likelihood MLST tree based on the concatenation of seven housekeeping genes (*atpD*, *clpB*, *gapA*, *gyrB*, *nuoD*, *pyrH* and *rpoB*). The phylogenetic sublineages are distinguished by color code (A: blue, B: red, C: green, D: yellow). Biosynthetic gene clusters, as detected by antiSMASH 3.0, are displayed for each strain.

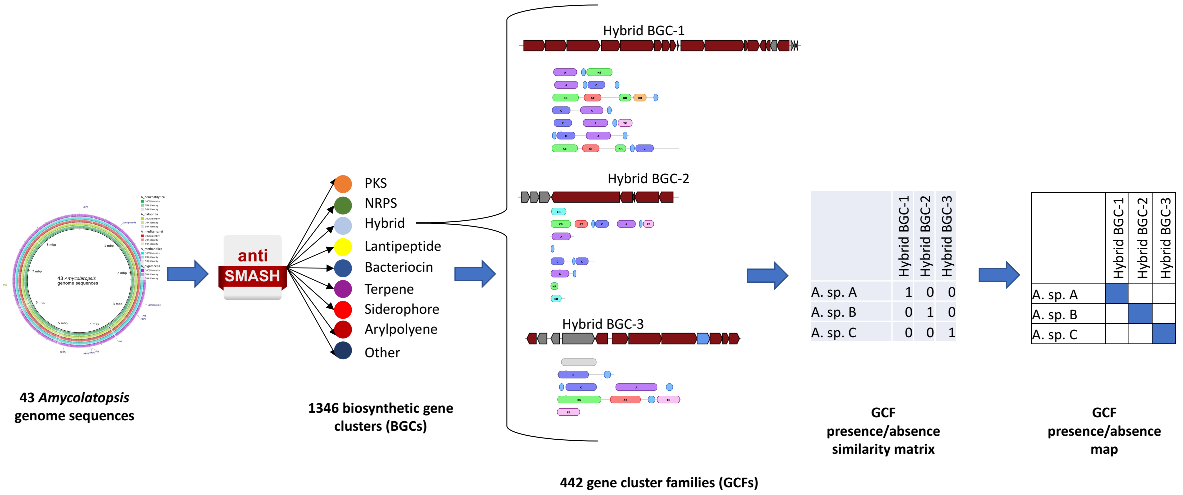

Figure S5: Rationale for the manual sorting of BGCs to cluster families. After first classification using the antiSMASH cluster definitions, BGCs were sorted according to their overall cluster architecture, where the majority of genes shared >50% Blast similarity over 80% sequence coverage. For PKS, NRPS and their hybrids additionally a similarity threshold of 80% Blast similarity of KS and/or C domains was used. Presence/absence of cluster families was collected in a similarity matrix, from which the presence/absence map in Figure 3 was derived.

	Gene cluster families (GCFs)			
Cluster type	in ≧ 4 strains	in 2-3 strains	unique	total
PKS	14	18	96	128
Hybrid	4	11	27	42
NRPS	16	15	54	85
RiPP	12	13	43	68
Terpene	10	2	21	33
Other	19	14	53	86
Sum BGC-families total	75	73	294	442

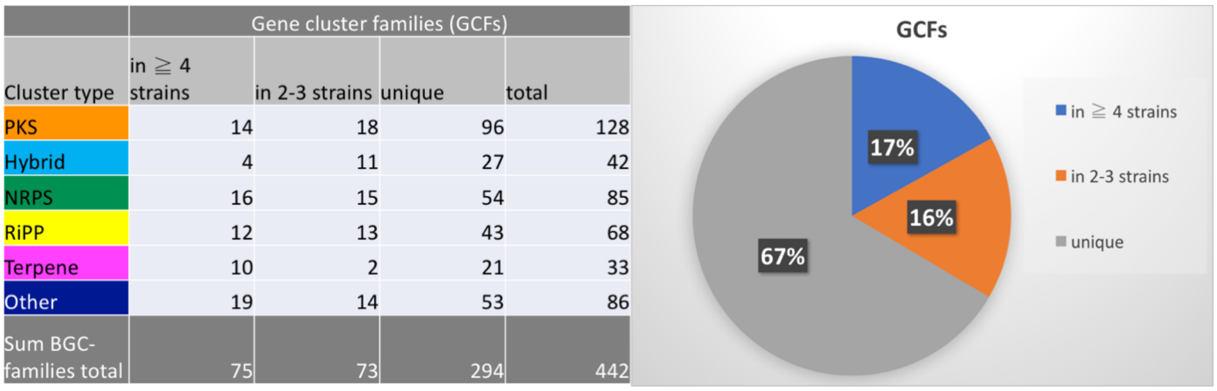

Figure S6: Total number of GCFs that are common (present in ≥4 strains), rare (present in 2-3 strains) and unique (present in only one strain) for 43 *Amycolatopsis* strains.

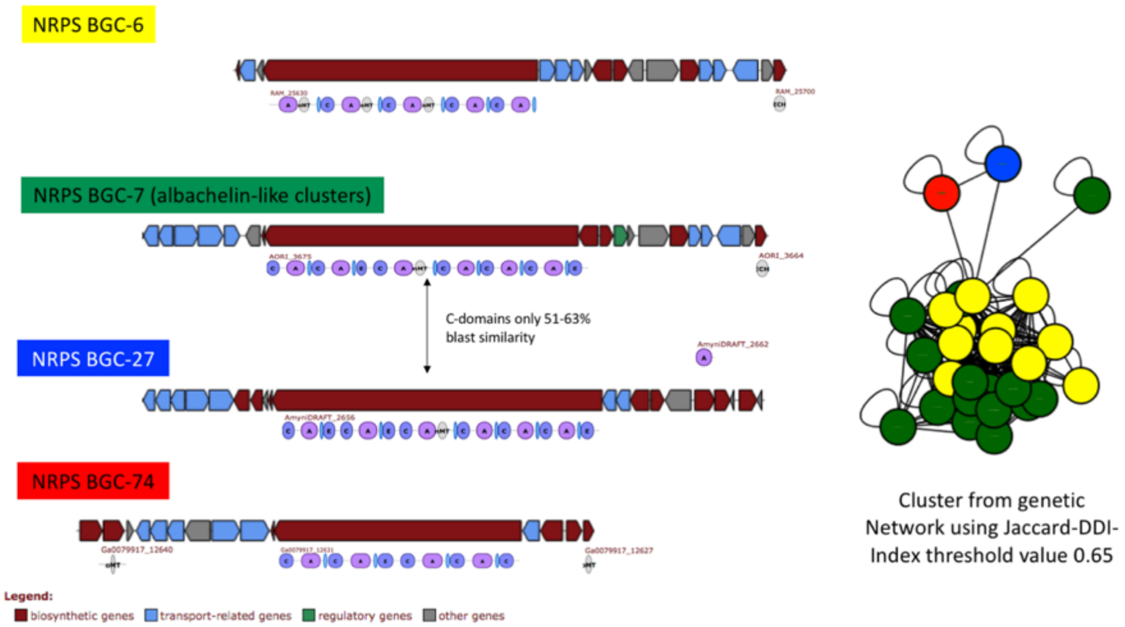

Figure S7: Example for four NRPS-BGCs that were categorized as four different GCFs, but cluster together within the genetic network calculated from the Jaccard-DDI-Index with a threshold of 0.65. With a higher threshold, these GCFs would form separate clusters, at the cost of disrupting other clusters with only few Pfam domains.

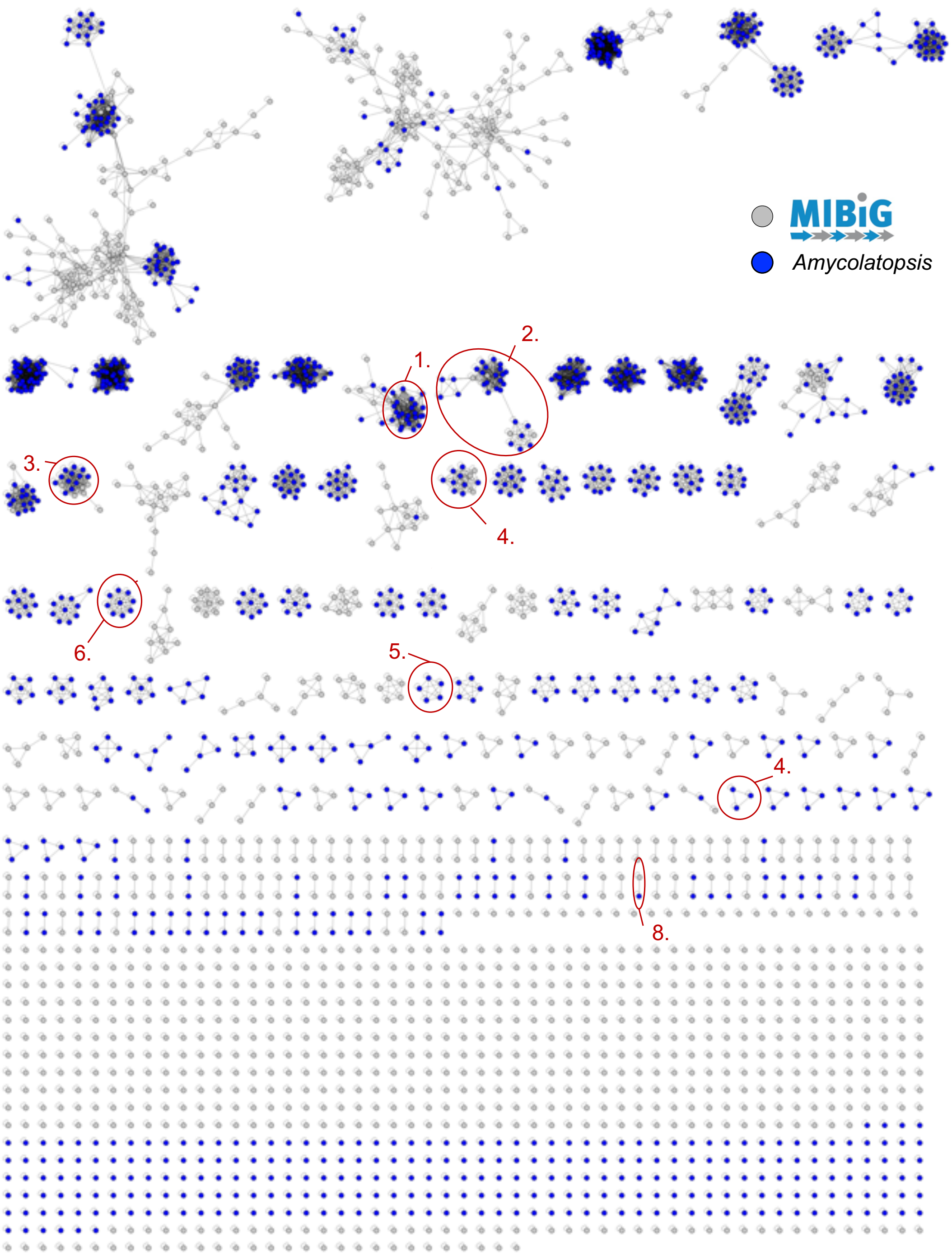

Figure S8: Genetic network of *Amycolatopsis* BGCs and known BGCs from the MIBiG database. Clustering was based on the comparison of Pfam-domains using the Jaccard/DDI index with a threshold of 0.65. Nodes representing BGCs from *Amycolatopsis* are colored in blue, nodes representing BGCs from the MIBiG database are colored in gray. 1. albachelin-like NRPS, 2. 2-methylisoborneol, 3. glycopeptides, 4. rifamycin, 5. ECO-0501, 6. macrotermycin-like PKS clusters, 7. octacosamicin, 8. chelocardin.

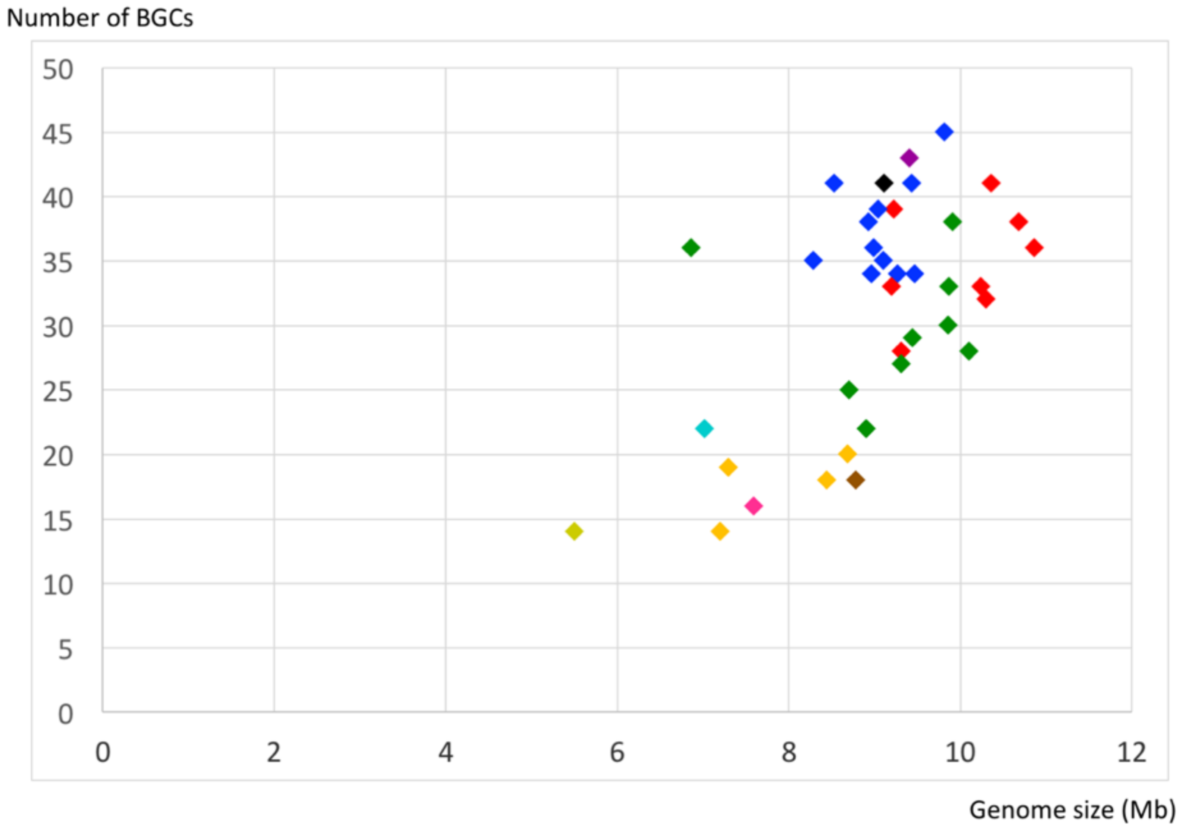

Figure S9: Genome size plotted against the number of BGCs. Colors represent the phylogenetic affiliation: blue (group A), red (group B), dark green (group C), yellow (group D), light green (*A. halophila*), turquoise (*A. marina*), purple (*A. xylanica*), pink (*A. sacchari*), brown (*A. taiwanensis*) and black (*A. nigrescens*).

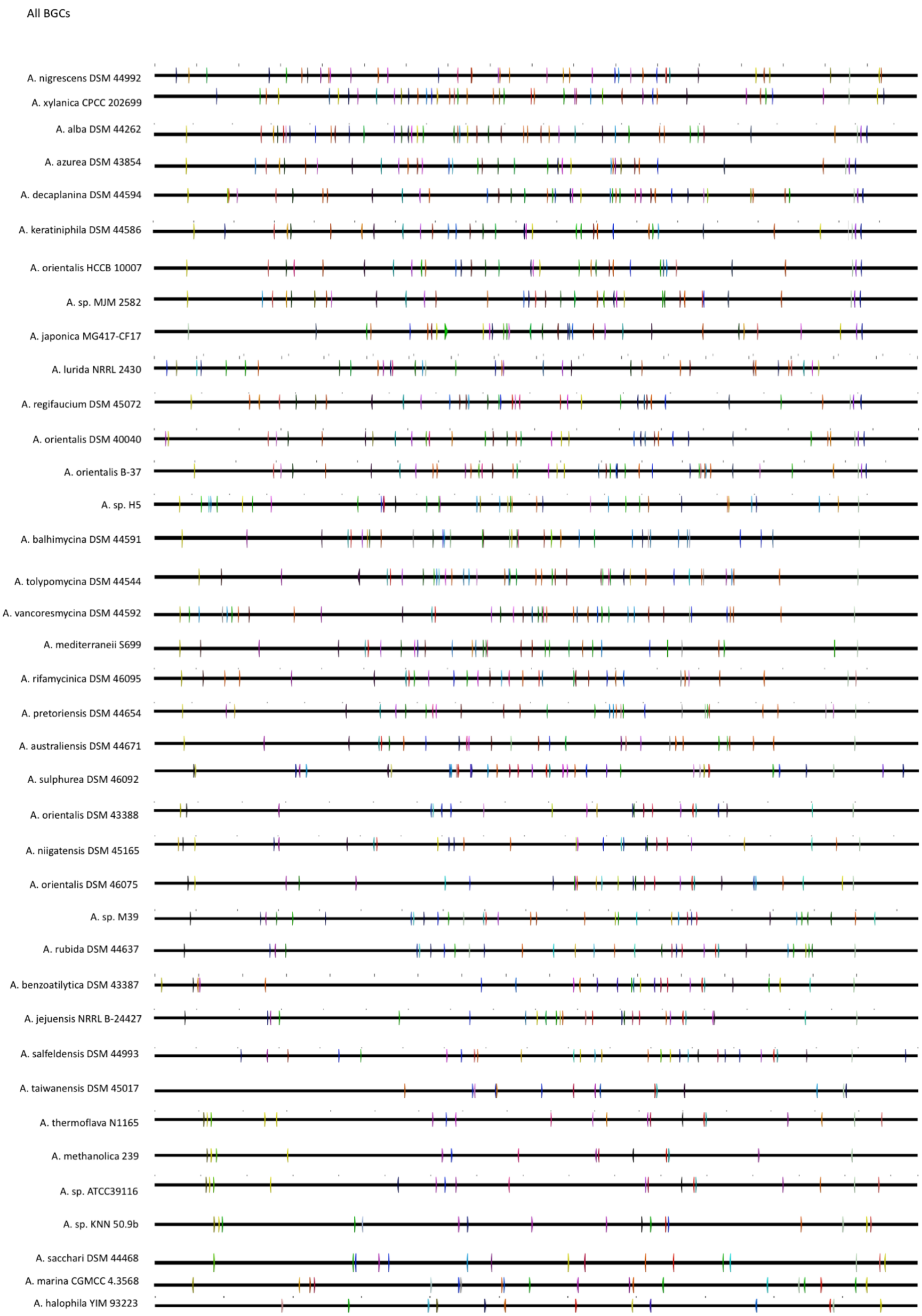

Figure S10: Location of all BGCs on *Amycolatopsis* genomes and pseudocontigs.

All BGCs

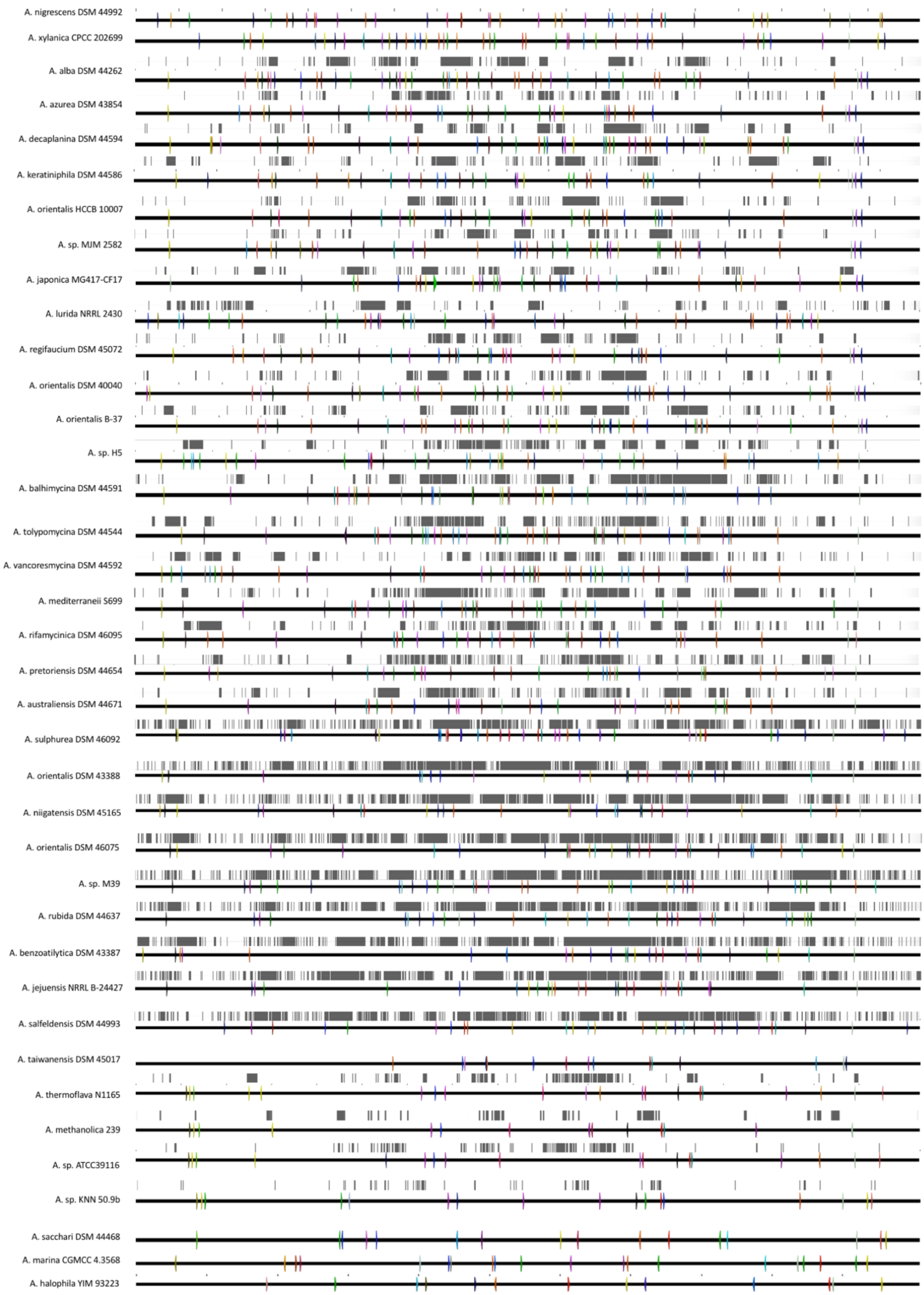

Figure S11: Location of all BGCs on *Amycolatopsis* genomes and pseudocontigs with the corresponding hypervariable regions highlighted in grey.

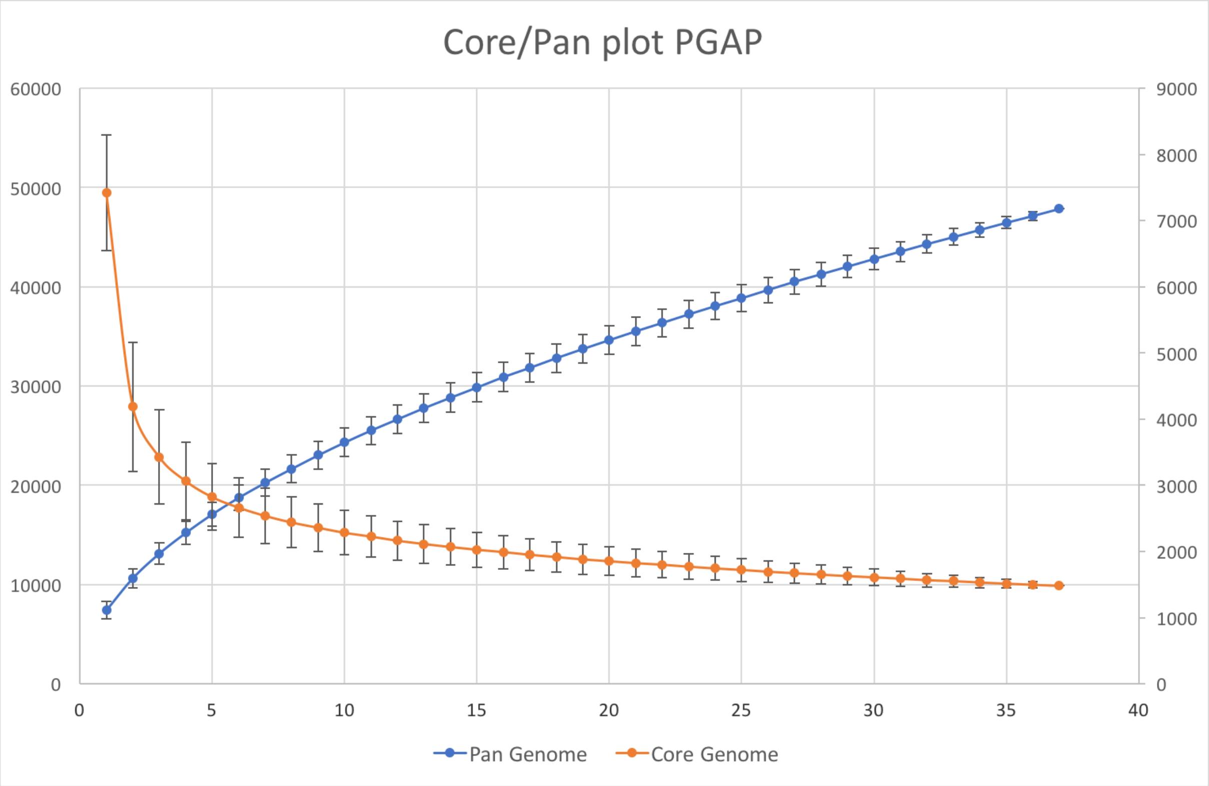

Figure S12: Core-/Pan-Genome plot for the genus *Amycolatopsis*. Analysis was performed using PGAP in the GF mode. The number gene families is plotted against the number of genomes added to the analysis. The left axis (blue curve) is representing the pan-genome with 47,846 genes from 37 analyzed genomes. The right axis (orange) is representing the core-genome with 1480 genes from 37 analyzed genomes.

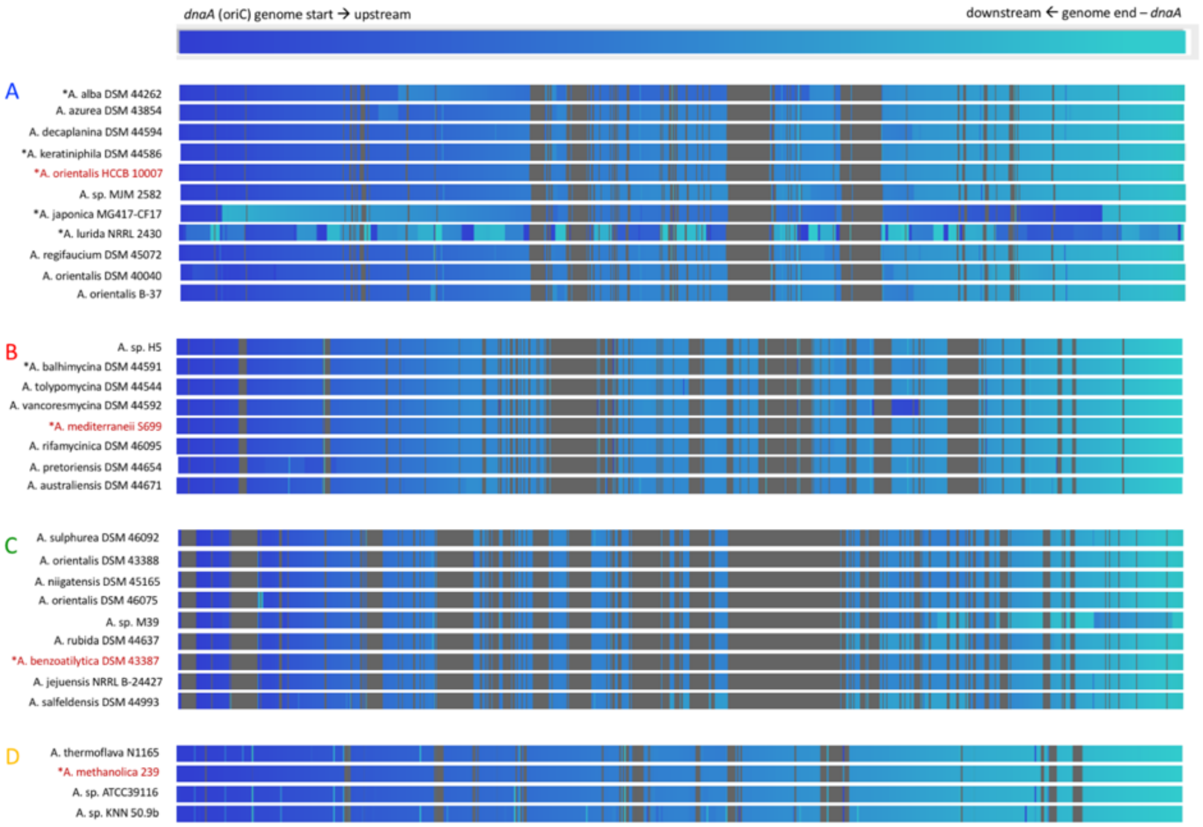

Figure S13: Genome synteny of complete genomes and pseudocontigs based on a core genome alignment for a reference strain in each phylogenetic group. Reference strains are highlighted in red. Unmapped regions specific for the reference strains are displayed in grey. Complete genomes and draft genomes with only one scaffold are highlighted with an asterisk (*).
