# Supplementary material for: Comparative genomics reveals phylogenetic distribution patterns of secondary metabolites in Amycolatopsis species
Source: BMC Genomics. 2018 Jun 1;19:426. doi: 10.1186/s12864-018-4809-4 (PMC5984834; doi:10.1186/s12864-018-4809-4)
Supplement: Supplementary file 2 — Table S1. Sequencing statistics for Amycolatopsis sp. H5 and Amycolatopsis sp. KNN50.9b. (DOCX 42 kb) [file 12864_2018_4809_MOESM2_ESM.docx]

**Supplementary Tables**

**Table S1**: *De novo* sequencing and assembly of *Amycolatopsis* sp. H5 and *Amycolatopsis* sp. KNN50.9b.

|  | ***Amycolatopsis* sp. H5** | ***Amycolatopsis* sp. KNN50.9b** |
| --- | --- | --- |
| Assembled reads | 4,785,333 | 5,728,135 |
| Assembled bases | 1,034,006,641 bp | 1,325,350,020 bp |
| Total sequence length | 10,673,263 bp | 7,317,183 bp |
| Coverage | 97x | 139x |
| GC content | 70.84% | 73.14% |
| Contigs | 183 | 287 |
| Max contig length | 604,881 bp | 338,492 bp |
| N50 | 236,110 bp | 101,997 bp |
