# Supplementary material for: Comparative genomics reveals phylogenetic distribution patterns of secondary metabolites in Amycolatopsis species
Source: BMC Genomics. 2018 Jun 1;19:426. doi: 10.1186/s12864-018-4809-4 (PMC5984834; doi:10.1186/s12864-018-4809-4)
Supplement: Supplementary file 3 — Table S2. Basic features of Amycolatopsis genomes. (DOCX 110 kb) [file 12864_2018_4809_MOESM3_ESM.docx]

**Table S2**: Basic features of *Amycolatopsis* genomes

| **Organism name** | **Group** | **Isolation site/ Sample type** | **Core genes** | **Accessory genes** | **Unique genes** | **Exclusively absent genes** | **Total number of genes** | **Number of BGCs** | **Genome size** | **Sequencing status** |
| --- | --- | --- | --- | --- | --- | --- | --- | --- | --- | --- |
| *A. nigrescens* DSM 44992 | no | Wall of an arcosolium in the catacomb St. Callistus; Rome, Italy | 1212 | 4816 | 2057 | 14 | 8085 | 41 | 9.11 Mb | complete |
| *A. xylanica* CPCC202699 | no | Soil, Qinghai province, China | 1212 | 4964 | 1939 | 6 | 8115 | 43 | 9.41 Mb | 25 scaffolds from 26 contigs |
| *A. alba* DSM 44262 | A | Soil, geographic region unknown | 1212 | 6599 | 712 | 1 | 8523 | 45 | 9.81 Mb | 1 scaffold from 2 contigs |
| *A. azurea* DSM 43854 | A | Soil, Japan | 1212 | 6570 | 430 | 2 | 8212 | 39 | 9.22 Mb | 154 contigs |
| *A. decaplanina* DSM 44594 | A | Soil, India | 1212 | 5921 | 387 | 8 | 7520 | 41 | 8.53 Mb | 85 contigs |
| *A. japonica* M417-CF17 | A | n.a. | 1212 | 6611 | 246 | 1 | 8069 | 34 | 8.96 Mb + 0.092 Mb plasmid | complete |
| *Amycolatopsis* sp. MJM2582 | A | Rhizosphere sample from Ami Mountain in Chungcheongnam-do province, Western part of South Korea | 1212 | 6531 | 253 | 2 | 7996 | 38 | 8.93 Mb | 13 scaffolds from 42 contigs |
| *A. keratiniphila* DSM 44586 | A | Soil, India | 1212 | 6799 | 353 | 1 | 8364 | 34 | 9.47 Mb | complete |
| *A. orientalis* HCCB10007 | A | Industrial strain derived from ATCC 43491 (from soil, East Indies) through physical and chemical mutagenesis | 1212 | 6477 | 204 | 2 | 7893 | 34 | 8.94 Mb + 0.033 Mb plasmid | complete |
| *A. lurida* NRRL 2430 | A | Soil, geographic region unknown | 1212 | 6446 | 443 | 1 | 8101 | 36 | 8.99 Mb | complete |
| *A. regifaucium* DSM 45072 | A | Arid soil, Kings Canyon, Australia | 1212 | 5741 | 398 | 0 | 7351 | 35 | 8.29 Mb | 49 contigs |
| *A. orientalis* DSM 40040 | A | Soil, geographic region unknown | 1212 | 6351 | 310 | 0 | 7873 | 35 | 9.1 Mb | 99 contigs |
| *A. orientalis* B-37 | A | Soil, Guizhou Province, China | 1212 | 6485 | 386 | 3 | 8083 | 41 | 9.43 Mb | 88 contigs |
| *A. australiensis* DSM 44671 | B | Composite soil, Australia | 1212 | 6284 | 784 | 0 | 8280 | 28 | 9.31 Mb | 10 contigs |
| *A. pretoriensis* DSM 44654 | B | Equine placenta; Pretoria, South Africa | 1212 | 7179 | 965 | 1 | 9356 | 32 | 10.30 Mb | 31 contigs |
| *A. mediterranei* S699 | B | Soil of pine arboretum at St. Raphael, France | 1212 | 6994 | 778 | 5 | 8984 | 33 | 10.24 Mb | complete |
| *A. rifamycinica* DSM 46095 | B | Soil, arid region near Alice Springs, Australia | 1212 | 6426 | 544 | 1 | 8182 | 33 | 9.20 Mb | 88 contigs |
| *A. tolypomycina* DSM 44544 | B | Soil, India | 1212 | 6841 | 825 | 1 | 8878 | 41 | 10,36 Mb | 4 contigs |
| *A. vancoresmycina* DSM 44592 | B | Soil, India | 1212 | 6662 | 634 | 3 | 8508 | 39 | 9.04 Mb | 82 contigs |
| *A. balhimycina* DSM 44591 | B | Soil, India | 1212 | 7061 | 902 | 7 | 9175 | 36 | 10.86 Mb | 1 scaffold from 10 contigs |
| *Amycolatopsis* sp. H5 | B | Gravel soil (30 cm depth), Cerro Chajnantor, a lava dome mountain in the Chilean Central Andes, Chile | 1212 | 7154 | 1043 | 1 | 9409 | 38 | 10.68 Mb | 183 contigs |
| *A. sulphurea* DSM 46092 | C | Garden soil | 1212 | 3530 | 1277 | 44 | 6019 | 36 | 6.86 Mb | 2 scaffolds |
| *A. orientalis* DSM 43388 | C | n. a. | 1212 | 6377 | 513 | 0 | 8102 | 22 | 8.9 Mb | 197 contigs |
| *A. niigatensis* DSM 45165 | C | Volcanic soil, Niigata, Japan | 1212 | 6526 | 595 | 2 | 8333 | 27 | 9.31 Mb | 3 scaffolds |
| *A. orientalis* DSM 46075 | C | Clinical isolate | 1212 | 6804 | 433 | 3 | 8449 | 29 | 9.44 Mb | 183 contigs |
| *Amycolatopsis*. sp. M39 | C | Termite tissue, South Africa | 1212 | 7035 | 618 | 3 | 8865 | 38 | 9.91 Mb | 63 scaffolds from 169 contigs |
| *A. rubida* DSM 44637 | C | Soil, conifer forest in Guangxi Province, China | 1212 | 7017 | 669 | 0 | 8898 | 33 | 9.87 Mb | 45 contigs |
| *A. benzoatilytica* DSM 43387 | C | Clinical sample, patient with submandibular mycetoma, Czechoslovakia | 1212 | 5628 | 827 | 1 | 7667 | 25 | 8.70 Mb | 1 scaffold from 6 contigs |
| *A. jejuensis* NRRL B-24427 | C | Dried bat dung, natural cave on Jeju Island, Republic of Korea | 1212 | 5224 | 2407 | 4 | 8843 | 28 | 10.10 Mb | 136 contigs |
| *A. saalfeldensis* DSM 44993 | C | Surfaces of acidic and heavy metal containing rocks, medieval alum slate mine, Germany | 1212 | 5786 | 1694 | 3 | 8692 | 30 | 9.86 mb | 66 scaffolds from 68 contigs |
| *Amycolatopsis* sp. 75iv2 | D | Soil, Idaho | 1212 | 6053 | 547 | 4 | 7812 | 18 | 8.44 Mb | 119 contigs |
| *A. thermoflava* N1165 | D | Soil, Hainan Island, China | 1212 | 6039 | 838 | 3 | 8089 | 20 | 8.69 Mb | 2 scaffolds from 7 contigs |
| *A. methanolica* 239 | D | Soil, New Guinea | 1212 | 5056 | 603 | 1 | 6871 | 14 | 7.20 Mb | complete |
| *Amycolatopsis* sp. KNN 50.9b | D | Halite encrusted soil (top 2 cm), edge of the Laguna Chaxa which is located within the Salar de Atacama, Chile | 1212 | 4916 | 690 | 2 | 6818 | 19 | 7.3 Mb | 287 contigs |
| *A. sacchari* DSM 44468 | no | Vegetable matter | 1212 | 4599 | 1224 | 10 | 7035 | 16 | 7.59 Mb | 72 contigs |
| *A. taiwanensis* DSM 45017 | no | Soil, Yilan, Taiwan | 1212 | 4292 | 2389 | 52 | 7893 | 18 | 8.78 Mb | 73 scaffolds from 93 contigs |
| *A. marina* CGMCC4.3568 | no | Ocean-sediment sample collected from the South China Sea | 1212 | 3541 | 1534 | 11 | 6287 | 22 | 7.02 Mb | 59 scaffolds from 64 contigs |
| *A. halophila* YIM 93223 | no | Salt lake in Xinjiang Province, north-west China | 1212 | 1739 | 1891 | 253 | 4842 | 14 | 5.5 Mb | 1 scaffold from 5 contigs |
